# Chromatin insulators *homie* and *nhomie* can interact with distant copies either together or separately, with distinct outcomes for enhancer-promoter interactions

**DOI:** 10.1371/journal.pgen.1011940

**Published:** 2026-06-05

**Authors:** Miki Fujioka, Wenfan Ke, Paul Schedl, James B. Jaynes

**Affiliations:** 1 Department of Biochemistry and Molecular Biology, Thomas Jefferson University, Philadelphia, Pennsylvania, United States of America; 2 Department of Molecular Biology, Princeton University, Princeton, New Jersey, United States of America; The University of Tokyo: Tokyo Daigaku, JAPAN

## Abstract

Chromatin insulators, a.k.a. boundary elements, separate regions of the chromosome with distinct chromatin characteristics, including distinct histone modifications. This activity affects gene expression by allowing chromatin domains to be stably regulated and maintained. Insulators also block enhancer-promoter interactions and, somewhat paradoxically, facilitate other interactions, particularly when they stitch together distant regions of the chromosome by pairing with specific partners. Here we explore how long-range interactions facilitated by insulator pairing are affected by the presence of two potentially competing partners. Our results show that when two partners are present, they can reduce each other’s effects on distant gene expression, suggesting that enhancer-promoter interactions are best facilitated by pairwise insulator interactions. When a distant copy of an *eve* insulator (*homie* or *nhomie*) is present, it can interact with either or both endogenous insulators. But when one endogenous insulator is removed, the remaining one interacts more strongly with the transgenic copy, biasing the induced enhancer-promoter interactions toward those nearest the remaining endogenous insulator. On the other hand, physical interaction data suggest that strictly pairwise interactions are not the rule, suggesting a more complex model involving tripartite interactions. We further show that removing one or both endogenous *eve* insulators significantly reduces endogenous *eve* function at a critical early stage of development, and that the *eve* Polycomb domain expands in both directions when its insulator boundaries are removed, showing that insulators in their native context are required for each of the main functions that have been ascribed to them based on transgene assays.

## Author summary

Chromatin insulators regulate gene activity and other nuclear functions by defining the end points of functionally separate regions of the chromosome. In order to do this, they physically interact with each other, forming structures whose properties are just beginning to be understood. Insulators block enhancer-promoter interactions and, paradoxically, facilitate other interactions, as they stitch together distant regions of the chromosome by pairing with specific partners. Here, we explore the architecture of insulator interactions by changing the number of compatible partners that are present. A distant copy of an *eve* insulator (*homie* or *nhomie*) can interact with either or both endogenous insulators. But when one endogenous insulator is removed, the remaining one interacts more strongly with the transgenic copy, affecting both enhancer-promoter and physical interactions. The results suggest a competition between different pairwise combinations, but also challenge the simplest version of a strictly pairwise model, in favor of a more complex, multi-component complex. We further show that endogenous *eve* insulators support full *eve* function, while they also constrain the spreading of the *eve* Polycomb domain to promote expression of adjacent genes.

## Introduction

Chromatin insulators are one of several elements that influence chromosome architecture by physically interacting with each other (pairing activity). In addition to insulators, Polycomb response elements (PREs) [1–5], as well as enhancers and promoters and their tethering elements, can be physically linked to each other, and so alter the topology of the chromatin fiber. Besides organizing chromosomes into topologically associating domains (TADs), insulators also impact gene expression. They can block enhancers from regulating the expression of a target gene when the potentially interacting elements are on opposite sides of them (enhancer blocking activity). Insulators also have a barrier activity that tends to block the spread of repressive chromatin such as Polycomb-modified chromatin and heterochromatin [6–10]. We recently showed that the endogenous *eve* insulator *homie* has multiple sub-domains that can differentially influence these various activities [11], suggesting that they may involve mechanisms that have both overlapping and distinct aspects.

The mechanisms involved in the various insulator activities intersect with their ability to physically pair with each other or with copies of themselves in cis or in trans. While insulators can often pair with a variety of different partners, they also have distinct partner preferences. These partner preferences are evident in both insulator competition assays [12] and insulator bypass assays [13–15]. Insulator pairing can also be orientation dependent, and the pairing can either be head-to-head or head-to-tail [16]. The self-pairing of Drosophila insulators is generally head-to-head, and evidence suggests that such interactions are largely responsible for aligning the homologs in register and holding them together [17–20]. Unlike self-pairing, heterologous pairing interactions can be either head-to-head or head-to-tail. The difference in pairing orientation determines whether the topology of the chromosome in the vicinity of the paired insulators has a circle-loop topology or a stem-loop topology.

In principle, insulators could interact with each other in more than one way, with different consequences for gene expression. First, because there are two copies of each DNA sequence near each other during G2, on the paired sister chromatids, these would be expected to interact with each other, holding sister chromatids together in register. Furthermore, when homologous chromosomes are paired, as they are throughout most of the Drosophila life cycle, there are 4 copies of each insulator near each other in G2. We know that paired homologs undergo transvection [21,22], in which enhancers on one homolog affect gene expression on the other, and that insulators pairing with each other can mediate this effect [17,23]. This suggests that multiple copies of a self-pairing insulator interact with each other in trans, at the same time that they interact with other, nearby or distant, insulators in cis. On the other hand, having more than one potential partner may result in a “time-sharing” competition between alternative partners. When and if more than two insulators interact with each other at the same time, these interactions could be cooperative. Alternatively, only pairwise interactions may occur at any given time, so that multiple potential partners may compete with each other. The question of how the various insulator activities are affected by pairing with multiple partners in cis has not been extensively investigated, and it is unclear whether phenomena like insulator bypass [13–15] and insulator competition [12] involve complex multi-partner interactions, or whether the effects are dependent on strictly pairwise interactions.

**Fig 1:**
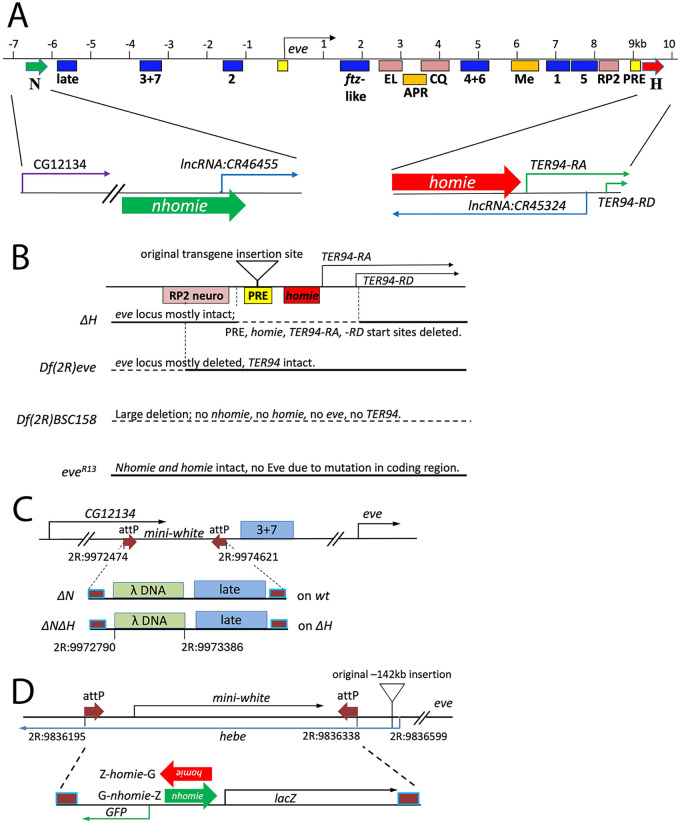
Maps of modified chromosomes and transgenes used in this study. **A.** Map of the *eve* locus. Boxes indicate locations of enhancers. Blue: stripe enhancers (early stripes: 3 + 7, 2, 4 + 6, 1, 5; the 7 late stripes: late; *ftz*-like stripes: *ftz*-like) [32,34–38], pink: neuronal enhancers (EL, CQ, and “RP2”, which drives expression in RP2 + a/pCC cells) [32,34], orange: anal plate ring (APR) and mesodermal (Me) enhancers [32,34], yellow: PREs [74]. Green block arrows: *nhomie* [17]. Red block arrows: *homie* [17,24]. Start sites and directions of transcripts are shown as thin arrows. **B.** Maps of mutant chromosomes used in this study. In each deficiency, a dotted line indicates a deleted region, while a solid line indicates an existing region. *∆H*: small deletion (2R: 9987987..9989353 is deleted) caused by imprecise excision of a homed P-element transgene (insertion site: triangle). It removes the *eve* 3’ PRE, *homie*, and the start sites of the *TER94-RA* and -*RD* transcripts. *Df(2R)eve* [33,41,55]: the deletion’s 5’ end is in *Mef2*, and its 3’ end is in the middle of the RP2 neuronal element (2R: 9949630.. 9987407 is deleted, see sequence in S1 Fig). Most of the *eve* locus is deleted, but *TER94* is intact. *Df(2R)BSC158*: large deletion (2R:9875312..10025310 is deleted) [39,40]. The *eve* and *TER94* loci are removed. *eve*^*R13*^ [41]: an amorphic point mutation in the *eve* coding region, which causes premature termination of the Eve protein [32]. **C.** Map of *∆N*, the *nhomie* deletion. The top line shows the attP insertion created using CRISPR. The *mini-white* gene was then replaced by the DNA fragment shown below, using RMCE. The 600 bp *nhomie* region was replaced by a similar-sized fragment of λ DNA (2R: 9972790..9973386 is deleted) on either a wild-type (creating *∆N*) [25] or the *∆H* chromosome (creating *∆N∆H*). The end points used to create the deletion are shown on the map. Brown square with blue outline: attP/attB fusion sequence left after RMCE [66]. **D.** Maps of reporter transgenes. Top map shows the attP-carrying chromosome used for RMCE. The original insertion site at –142kb [17,24] is shown as a triangle (2R:9836599). Dual reporter genes carrying either *homie* (Z-*homie*-G) or *nhomie* (G-*nhomie*-Z) replaced *mini-white*. *homie* and *nhomie* in the transgene are oriented so that *lacZ* is more strongly expressed when they interact orientation-specifically with the *eve* locus (see text).

Here, we use both functional assays, based on reporter gene activity that depends on the tethering of distant enhancers to the promoter, along with physical interaction assays (MicroC), to investigate this issue. In previous studies, we used a long-range assay to investigate the physical pairing activities of the two insulators, *nhomie* and *homie*, that flank the *even-skipped* (*eve*) locus [11,17,24]. We established that the endogenous *eve* insulators interact with each other in a specific orientation (head-to-tail) [25], and that they can also interact with transgenic copies of themselves and each other from considerable distances away along the chromosome. In the reporter gene assay, a transgene carrying two divergently transcribed reporters, *lacZ* and *gfp*, each under the control of an *eve* promoter, is inserted 142 kb from the *eve* gene. When either *nhomie* or *homie* is included between the dual reporters, it physically pairs with the endogenous *eve* locus, across the dozen or so TADs in between, and this brings the transgene into proximity to the *eve* enhancers. Because their pairing is orientation-dependent, one of the two reporters is activated much more strongly by the *eve* enhancers, and this depends on the orientation of the insulator in the transgene. It does not, however, depend on the orientation of the dual reporter transgene in the chromosome [17]. In the *eve* locus, the *eve* transcription unit is downstream of *nhomie* and upstream of *homie*, based on the direction of transcription of *eve* (Fig 1A). This same configuration rule applies to the transgene insulator: the reporter that is downstream of *nhomie* is activated by the *eve* enhancers, while the reporter upstream of *homie* is the one primarily activated. Orientation-dependent physical interactions between sequences in the transgene and the *eve* locus, detected using the MicroC procedure, reflect these regulatory interactions [25,26]. When the transgene containing *nhomie* or *homie* is inserted at the -142kb site, transgene reporter expression is limited to a small percentage of cells within the stripes, instead of being uniform throughout each stripe [17,24]. Consistent with this, a previous live imaging study indicated that the transgene is only in contact with the endogenous *eve* locus in a subset of nuclei [27].

While the long-range interactions between *nhomie-* or *homie*-containing transgenes and the *eve* locus are artificial, recent studies using MicroC have shown that some endogenous insulators separated by dozens of intervening TADs can find each other and physically pair [28,29]. Moreover, like for *nhomie* and *homie*, these long-range interactions are both specific, and orientation-dependent. However, most of these naturally occurring loops differ from the artificial interactions studied previously in that only two insulators, one at each end of the loop, are typically involved, whereas the transgene *nhomie* or *homie* has two potential pairing partners in the *eve* locus. This may result in a more complicated set of regulatory and physical interactions. In this study, to better understand the differences between one and two potential pairing partners, we generated deletions of endogenous *nhomie* (*∆N*), endogenous *homie* (*∆H*), and simultaneous deletion of both (*∆N∆H*). These deletions were combined with the dual reporter transgene carrying either *nhomie* or *homie*. We find that the pairing interactions between the transgene insulator and the remaining endogenous insulator are substantially different from those observed with the wild-type *eve* locus. We also show that *eve* function in the *∆N* and *∆H* deletions, as well as in *∆N∆H*, is reduced. In addition to reducing *eve* function, these insulator deletions result in the spread of PcG-dependent histone modifications into the neighboring TADs, showing that one of the key activities of insulators in their endogenous locations is the establishment of units of independent gene regulation.

## Results

### Long-range insulator interactions can facilitate locally biased enhancer-promoter interactions

Previous studies showed that *homie* and *nhomie* can mediate long-range regulatory interactions when included in a reporter transgene that is inserted within several Mb of the *eve* locus [17,24]. Because the pairing interactions of the *eve* insulators are orientation-dependent, one of the two reporters in a dual reporter transgene is preferentially activated by the *eve* enhancers [17]. Although there are *eve* enhancers located both upstream (on the *nhomie* side) and downstream (on the *homie* side) of the *eve* transcribed region (Fig 1A), there is no apparent bias in their ability to activate reporter expression. This is reflected in the pattern of physical contacts with the transgene seen in MicroC experiments, which extend across the entire *eve* locus, with peaks at the insulators, *nhomie* and *homie* [26]. One interpretation of the MicroC data is that the transgene insulator interacts simultaneously with endogenous *homie and nhomie*, forming a tripartite complex. An alternative possibility is that there are three different pairwise interactions, transgene insulator with either endogenous *nhomie* or *homie*, and endogenous *nhomie* with *homie*, generating three different types of loops that potentially compete with each other. This led us to test how the transgene would interact with the *eve* enhancers if there were only one endogenous *eve* insulator.

### Endogenous *homie* and *nhomie* are required for the long-range interaction

To answer this question, we first deleted either endogenous *nhomie* or *homie*. We used CRISPR [30,31] to remove endogenous *nhomie* (creating *∆N*; see Fig 1C for map) [25], but were unsuccessful at obtaining a similarly “clean” removal of endogenous *homie*. However, we were able to obtain a small deletion that includes endogenous *homie*, along with the 3’ *eve* PRE and the *TER94* basal promoter region and first exon (Fig 1B; see S1 Fig for sequence). This mutant chromosome (*∆H*) is homozygous lethal after embryogenesis. In earlier studies, we showed that two copies of a transgenic *eve* rescue construct that did not include *homie* were able to rescue the lethality of *eve* homozygous null mutants (both *eve*^*R13*^*/eve*^*R13*^ and *eve*^*R13*^*/Df(2R)eve*) [32]. This rescue construct contained the *eve* locus from –6.4 to +8.4 kb (relative to *eve* + 1 transcription start site), which included all of the *eve* enhancers (the 3’-most of which is the RP2 neuronal element), but not *nhomie* or *homie*. The 3’ endpoint of this rescue construct (2R:9987733) is close to the 5’ endpoint of the *∆H* deletion (2R:9987987), which also retains this neuronal element but is missing *homie*. Therefore, it seems likely that the homozygous *∆H* chromosome is able to provide sufficient *eve* function for full rescue of viability, and so the loss of *eve* function is not the cause of the *∆H* lethality. In order to test whether the lethality of *∆H* comes instead from removal of the *TER94* promoter, we complemented it with an *eve*-deficient chromosome, *Df(2R)eve*, carrying an intact *TER94* locus. To do this, we first confirmed that *Df(2R)eve* is a complete null for *eve* function [33], but has intact *TER94* function (Fig 1B; see S1 Fig for junction sequence). When the *∆H* chromosome was placed over *Df(2R)eve*, some adult flies survived. Some of the surviving adults showed abnormal segmentation that is not evident in *Df(2R)eve* heterozygotes. Nonetheless, rescue to adulthood by *Df(2R)eve* indicates that the recessive lethality of *∆H* is mainly caused by lack of *TER94* function. Segmentation defects in *∆H/Df(2R)eve* adult flies suggest that the *eve* function provided by *∆H* is significantly weakened, and this is confirmed below by analyzing embryonic defects. For further analysis, we generated a chromosome lacking both *homie* and *nhomie* (*∆N∆H*), replacing *nhomie* on the *∆H* chromosome with *lambda* DNA, using CRISPR (Fig 1C; see also Materials and Methods). The fertility of *∆N∆H/Df(2R)eve* seems to be lower than that of *∆H/Df(2R)eve*, as we could maintain *∆N∆H/Df(2R)eve* for only a few generations.

In order to analyze interactions between transgenes and the modified endogenous *eve* locus, we used CRISPR to insert a pair of attP sites (flanking a *mini-white* marker gene) onto the wild-type (wt), *∆N*, *∆H,* and *∆N∆H* chromosomes, near our previously used attP site (142kb upstream of *eve*). The *mini-white* marker was then replaced with our *lacZ-gfp* dual reporter transgene vector [11,17] (Fig 1D). In previous studies [17,26], we showed that transgene activation is biased according to how *homie* or *nhomie* is oriented in the transgene relative to the two reporter genes. The reporter located either upstream of *homie* or downstream of *nhomie* is preferentially activated by the *eve* enhancers (see Fig 1A and 1D; red or green arrow shows the orientation of *homie* or *nhomie*, respectively). Throughout this study, we used the two transgenes that preferentially express *lacZ*, which we call Z-*homie*-G and G-*nhomie*-Z. The negative control (*lambda* DNA in place of a transgenic insulator) did not show any *eve*-like *lacZ* expression (S2 Fig). However, various patterns of ectopic expression that we previously reported are seen [11]. S3 Fig shows positive control expression of each of the two reporters with *homie* and *nhomie* at the new attP site on a wild-type chromosome. In both lines, *lacZ* is expressed as 7 stripes at stages 5–9 (S3 Fig, stages 5 and 7 are shown), and in a tissue-specific *eve* pattern in the mesoderm, anal plate ring (APR), and central nervous system (CNS) at later stages (S3 Fig, stages 11 and 13 are shown). *gfp* is not expressed in the *eve* pattern, instead, ventral midline expression in the CNS driven by an enhancer near the insertion site is observed. In each case, these expression patterns match previously reported results with the same dual-reporter transgenes inserted at the original attP site [11,17].

**Fig 2:**
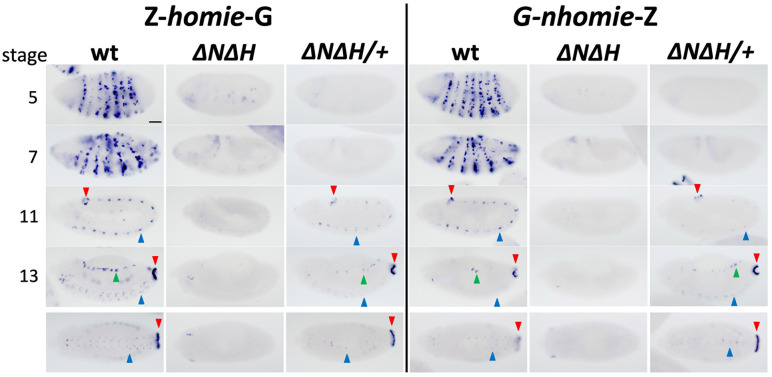
Transgenic *homie* and *nhomie* require endogenous *homie* and *nhomie* for long-range interaction with *eve* enhancers, which can occur in trans at later embryonic stages. RNA expression from *lacZ* reporter transgenes carrying *homie* (*Z-homie-G*) or *nhomie* (*G-nhomie-Z*) located at –142kb on homozygous wild-type (wt) or *∆N∆H* chromosomes, and heterozygous *∆N∆H* over wild-type (∆N∆H/ + , showing a *trans* interaction). Arrowheads indicate *eve* tissue-specific expression: red: APR; blue: CNS; green: mesoderm. Embryonic stages 5, 7, 11, and 13 are shown. Ventral views of stage 13 are shown in the bottom row. Scale bar: 50μm.

In previous experiments, we found that long-range (LR) interactions between reporters in the transgene and the *eve* enhancers only take place when either *homie* or *nhomie* is included in the transgene [11,17]. We concluded that the LR interaction between the *eve* locus and transgenic reporter genes is mediated by directional (orientation-specific) pairing between *homie* or *nhomie* in transgenes and their endogenous counterparts. Further analysis using live-cell imaging [27] and physical interaction studies using MicroC [26] were consistent with this explanation. This model predicts that the LR interaction will disappear without endogenous *homie* and *nhomie*. So, we tested whether removing both endogenous *homie* and *nhomie* (*∆N∆H*) causes loss of the LR interaction. As predicted, no *eve*-like *lacZ* expression was seen from either Z-*homie*-G or G-*nhomie*-Z when they were carried on the *∆N∆H* chromosome (Fig 2, compare wild type and *∆N∆H*). These findings indicate that physical pairing between the endogenous and transgenic copies of the *eve* insulators are required for the LR interaction.

To test this idea further we took advantage of the fact that once homologs in somatic cells pair with each other, trans-regulatory interactions (transvection) are observed if enhancers on one homolog are brought into close proximity with target genes on the other homolog. Thus, if insulators at the *eve* locus mediate interactions with the transgene, then it should be possible to restore *lacZ* expression with a wild-type *eve* locus is *trans* to either Z-*homie*-G,*∆N∆H* or G-*nhomie*-Z,*∆N∆H.* Homolog pairing is limited at the blastoderm stage, and we do not detect *eve*-like *lacZ* expression. However, later in development, during stages 11–13, the APR enhancer drives *lacZ* expression in the posterior, and *eve*-like expression is also detected in the CNS and mesoderm (Fig 2, compare *∆N∆H* and *∆N∆H/+*).

### Pairwise insulator interactions bias enhancer-promoter interactions

We next tested whether endogenous *homie* and *nhomie* individually can mediate the LR interaction with transgenic copies of either themselves or each other. To do this, *mini-white*-carrying attP sites were introduced on each of the *∆H* and *∆N* chromosomes, as described above (Fig 1D). At stage 5, each *eve* stripe is controlled by a distinct “early” stripe enhancer [32,34–38]. As diagrammed in Fig 1A, the enhancers for stripes 3 + 7 and 2 are located upstream of the *eve* transcription unit, while enhancers for stripes 4 + 6, 1, and 5 are located downstream. An imprecisely mapped stripe 1 enhancer or “influencer” near the 5’ end of the *eve* locus has also been reported (not included in Fig 1D) [38]. At stages 6–8, the 7 stripes are under control of a single “late stripe” enhancer located near *nhomie* [37,38]. Tissue-specific enhancers for APR, mesoderm, and CNS expression are located downstream of the *eve* transcription unit [32,34].

**Fig 3:**
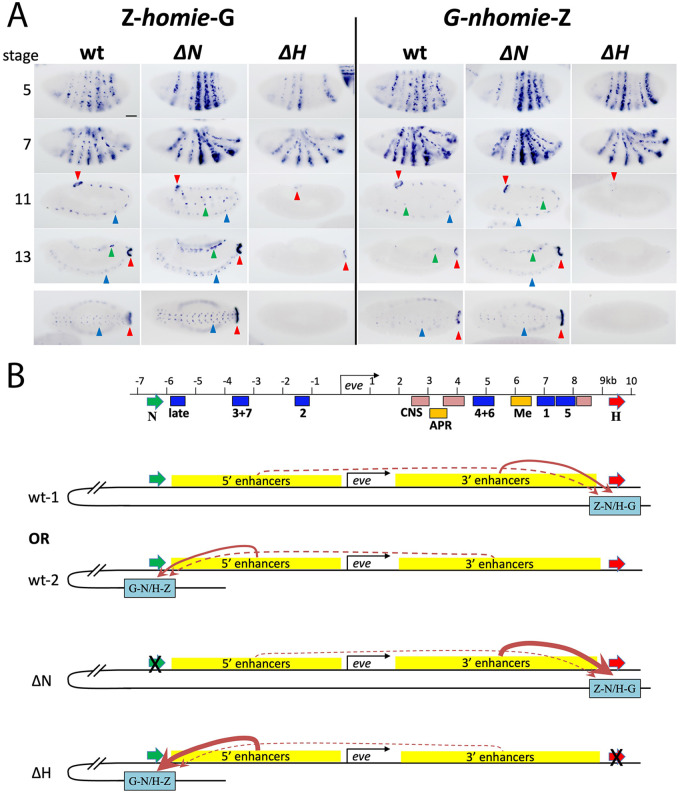
When one endogenous *eve* insulator is removed, long-range interactions become biased toward enhancers near the remaining endogenous insulator. **A.** RNA expression from *lacZ* reporter transgenes carrying *homie* (*Z-homie-G*, left panel) or *nhomie* (*G-nhomie-Z*, right panel) located at –142kb on homozygous wild-type (wt), *∆N*, or *∆H* chromosomes. Arrowheads indicate *eve* tissue-specific expression: red: APR; blue: CNS; green: mesoderm. Embryonic stages 5, 7, 11, and 13 are shown. Ventral views of stage 13 are shown in the bottom row. Scale bar: 50μm. **B.** Model for the pairwise LR interaction. **Top panel:** location of the *eve* enhancers. **Bottom 4 diagrams (simple pair-wise interaction model):** blue boxes represent the transgenes (Z-N/H-G: Z-*homie*-G or G-*nhomie*-Z at -142kb attP site), interacting with either *homie* (thick red arrow) or *nhomie* (thick green arrow) at the *eve* locus. Red curved arrows indicate activation of the *lacZ* transgene reporter by the *eve* enhancers. The thickness of these arrows indicates the strength/frequency of interaction. **wt:** *nhomie* or *homie* in the transgene interacts alternatively with either endogenous *nhomie* or *homie*. The 5’ enhancers activate *lacZ* when the transgene is paired with *nhomie*. Similarly, the 3’ enhancers activate *lacZ* when the transgene is paired with *homie*. **∆N:** *nhomie*/*homie* in the transgene interacts with endogenous *homie* more frequently (or more stably) than it does in wild type, where there is competition with endogenous *nhomie*. As a result, the 3’ enhancers activate *lacZ* more than in wild type. **∆H:** *nhomie*/*homie* in the transgene interacts with endogenous *nhomie* more frequently than in wild type, where there is competition with endogenous *homie*. As a result, the 5’ enhancers activate *lacZ* more than in wild type.

On either *∆N* or *∆H*, Z-*homie*-G and G-*nhomie*-Z each showed an LR interaction, expressing *lacZ* in a partial *eve* pattern. Importantly, however, these patterns differed from the interactions seen with the intact *eve* locus (Fig 3). In a wild-type background (Fig 3A and 3B, wt), at stage 5, the level of *lacZ* expression from either Z-*homie*-G or G-*nhomie*-Z is close to equal among the different stripes, except that the stripe 2 enhancer typically drives less *lacZ* expression than the others at this stage. This could be due to promoter competition, as the stripe 2 enhancer is located next to the endogenous *eve* promoter. At stage 7, the 7 stripes are also expressed at similar levels. All aspects of later-stage tissue-specific *eve* expression are also observed.

The pattern of *lacZ* expression in *∆N* differs from that in a wild-type background in that the level of *lacZ* expression in the seven stripes at stage 5 is much more uneven. With either Z-*homie*-G or G-*nhomie*-Z on *∆N* (Fig 3A and 3B, *∆N*), *lacZ* is expressed more strongly than in wild-type in stripes 4, 5, and 6. In contrast, expression is weaker than in wild-type in stripes 2, 3, and 7. At stage 7, the 7-stripe pattern is observed, but stripes 4, 5, and 6 appear stronger, likely because there are transcripts persisting from stage 5. The *eve* tissue-specific pattern at stages 11–13 in mesodermal, CNS, and APR is also observed, and the level of *lacZ* expression again appears stronger than when the *eve* locus is intact (Fig 3A, compare wild type and *∆N*). Strikingly, the enhancers for each of the aspects of the *eve* pattern that are stronger than in wild type are located 3’ of the *eve* transcription unit, closer to the remaining endogenous insulator, *homie*. However, there are two exceptions. First, although the precisely mapped stripe 1 enhancer is located near *homie*, stripe 1 expression was not as strong as that in stripes 4, 5, and 6. This is likely due to the location of the other stripe 1 element nearer *nhomie*, as noted above. Second, although the late-stripe enhancer is located near *nhomie*, this pattern appears to be less reduced than that of early stripes 2, 3, and 7. The likely explanation for this anomaly is a combination of proximity and competition with the endogenous *eve* promoter: in *∆N*, although the late-stripe enhancer is further from the tethering point of the transgene (at endogenous *homie*) than are the other enhancers (for early stripes 2, 3, and 7), there is also less competition with the endogenous *eve* promoter, which is further away. Therefore, the reduced competition outweighs the effect of the extra distance, making the late-stripe pattern (minus the extra contribution from the early stripes) more similar between *∆N* and wild type than is the early stripe 2, 3, and 7 expression pattern.

A reciprocal pattern of expression is observed for the transgene combinations with *∆H* (Fig 3A and 3B, *∆H*). At stage 5, *lacZ* expression from either Z-*homie*-G or G-*nhomie*-Z is stronger in stripes 2, 3, and 7, and weaker in stripes 4, 5, and 6, than they are in wild type. The late-stripe expression at stage 7 is similar to that seen in wild type. Furthermore, APR expression at stages 11–13 is less frequently observed, and then only faintly (Fig 3A, compare wild type and *∆H*). The enhancers for stripes 2, 3, and 7, as well as the late-stripe enhancer, are all located upstream of the *eve* promoter, closer to *nhomie*. Despite the location near *homie* of the mapped stripe 1 enhancer, stripe 1 expression is stronger than that of the other early stripes with enhancers located in the downstream region. As mentioned above, a second enhancer that contributes to stripe 1 expression was reported upstream of the *eve* promoter [38]. This can explain why stripe 1 does not behave like the other stripes that have enhancers only downstream of the *eve* promoter.

### Transgene – endogenous *eve* interactions: cis vs. trans

The distinct bias in reporter gene activity in either *∆H* or *∆N* raises an intriguing question: when *homie*- or *nhomie*-carrying transgenes engage in LR interactions in a wild-type background, do they pair with both of the endogenous insulators at the same time, or do they pair with only one of them at a time? The idea that interactions between insulators might be strictly pairwise would fit with results in which three different insulators were tested for partner preferences in an insulator competition assay [12].

**Fig 4:**
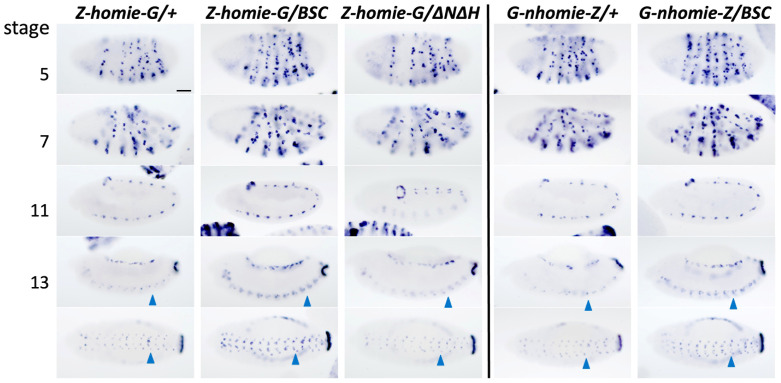
Late-stage interactions, especially in the CNS, become stronger without a trans copy of the *eve* locus. RNA expression from heterozygous *lacZ* reporter transgenes carrying *homie* (*Z-homie-G*, left panel) or *nhomie* (*G-nhomie-Z*, right panel) located at –142kb, in trans with either a chromosome carrying a wild-type *eve* locus (reporter transgene/+), *Df(2R)BSC158* (reporter transgene*/BSC*), or *∆N∆H* (*Z-homie-G/∆N∆H*). Embryonic stages 5, 7, 11, 13 are shown. Ventral views of stage 13 are shown in the bottom row. Note that *lacZ* expression, especially in the CNS at stage 13 (blue arrowheads), is stronger when the *eve* locus is absent in trans (compare *BSC* to *+*), but not when only the *eve* insulators are missing on the trans chromosome *(∆N∆H*). Scale bar: 50μm.

If insulator pairing interactions are exclusively pairwise, then there should be a competition between potential pairing partners when there are three or more of them. As a test of this idea, we manipulated the number of competing insulators by generating trans combinations between homologous chromosomes, one carrying the transgene and a wild-type *eve* locus, and the other carrying either a wild-type or mutant version of the *eve* locus. The *homie*- or *nhomie*-carrying transgene can interact with the endogenous *eve* locus either in cis or in trans (Fig 2), particularly at later embryonic stages, when wild-type homologs are fully paired. If there is a competition between the cis and trans interactions, then later-stage interactions might become stronger without a trans copy of the *eve* locus, resulting in stronger expression in the mesoderm, CNS, and APR. To remove the trans copy of endogenous *eve* completely, we used the large chromosomal deficiency *Df(2R)BSC158* (*BSC158*; see Fig 1B) [39,40]. When we compared *lacZ* expression in *Z-homie-G*/ + embryos with *Z-homie-G/BSC158*, we saw stronger expression with *Z-homie-G/BSC158* in the CNS over that seen with the wild-type chromosome (marked with *Sco*). This occurred with either *nhomie* or *homie* in the transgene (Fig 4: cis interaction only, 2^nd^ and 5^th^ columns, vs. in cis with a trans copy of *eve* on the *Sco*-carrying chromosome, 1^st^ and 4^th^ columns). At stages 5–7, the stripe expression may also be increased, which is consistent with some trans interactions occurring even at these early stages [23].

These results are consistent with the occurrence of trans competition between multiple possible pairwise interactions, but it is not the only possible explanation. To test specifically whether insulator pairing competition is responsible for this effect, we used the *∆N∆H* chromosome in trans to remove the potentially competing copies of the *eve* insulators. Here, when we compared *Z-homie-G/+* with *Z-homie-G/∆N∆H*, we no longer saw the increased expression (Fig 4, 3^rd^ column vs. 1^st^ column). These data indicate that the increased expression with *BSC158* is probably not due to competition between the transgene and the cis and trans copies of the endogenous insulators, but to a disruption of normal homolog pairing in *BSC158* heterozygotes (see Discussion).

### MicroC analysis of physical contacts between *homie*- and *nhomie-*containing transgenes and the *eve* locus

**Fig 5:**
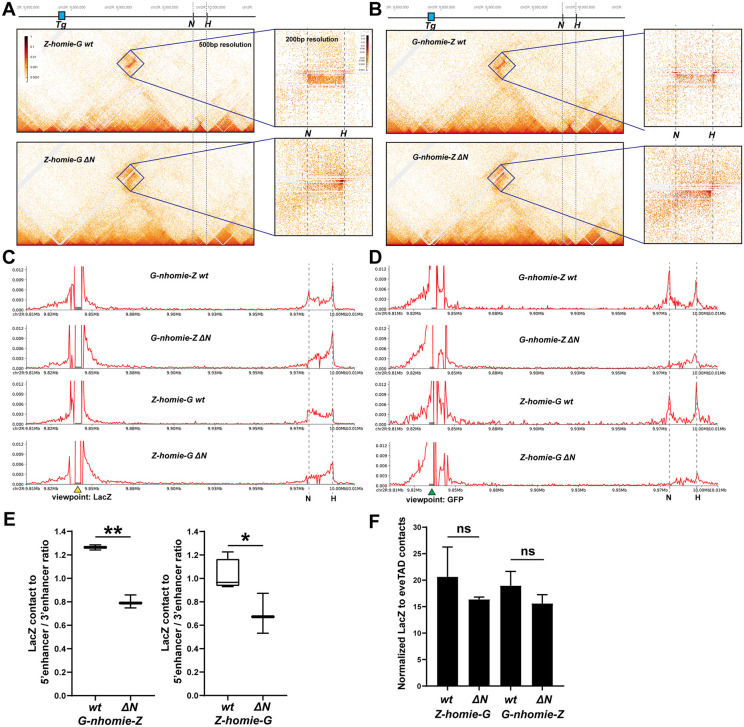
Physical interactions between the transgene and *eve* in wild type and *∆N.* **A.** MicroC contact profiles between *Z-homie-G* in wild type (top) and *∆N* (bottom). A blow-up of the interaction between the transgene and *eve* in each background is shown on the right of each panel. **B.** MicroC contact profiles between *G-nhomie-L* in wild type (top) and *∆N* (bottom). A blow-up of the interaction between the transgene and *eve* in each background is shown on the right of each panel. **C.** View points from the transgene *lacZ* reporter. From top to bottom, *G-nhomie-Z* in wild type, *G-nhomie-Z* in *∆N*, *Z-homie-G* in wild type, and *Z-homie-G* in *∆N*. **D.** View points from the transgene *gfp* reporter. From top to bottom, *G-nhomie-Z* in wild type, *G-nhomie-Z* in *∆N*, *Z-homie-G* in wild type, and *Z-homie-G* in *∆N*. **E.** Left panel: the ratio of contacts between *lacZ* and enhancers upstream (5’ enhancers) and downstream (3’ enhancers) of the *eve* gene in *G-nhomie-Z*, in a wild-type and a *∆N* background. Right panel: the ratio of contacts between *lacZ* and enhancers upstream (5’ enhancers) and downstream (3’ enhancers) of the *eve* gene in *Z-homie-G*, in a wild-type and a *∆N* background. **F.** Normalized contacts between *eve* and *lacZ* in either *G-nhomie-Z* or *Z-homie-G*, and in either wild type or *∆N*, as indicated.

In the *∆N* or *∆H* deletions, transgene reporter expression is preferentially activated by enhancers located on the same side of the *eve* transcription unit as the remaining endogenous insulator (Fig 3). This bias in reporter expression is strikingly reflected in the pattern of physical interactions between the transgene reporter and sequences in the *eve* locus (Fig 5). We used MicroC to analyze the physical contacts that are generated between each of the transgenes, G-*nhomie*-Z or Z-*homie*-G, and sequences in the *eve* locus, both in a wild-type *eve* background and in the *∆N* lines. Panels 5A-D show the MicroC contact profiles between the transgene and *eve*, while panels 5E and F show viewpoints from the transgene *lacZ* reporter. In a wild-type background, the *lacZ* sequences in Z-*homie*-G (Fig 5A, top panel and C, 3^rd^ panel) and G-*nhomie*-Z (Fig 5B, top panel and C, top panel) physically engage *eve* sequences located throughout the *eve* locus, with endpoint peaks that coincide with endogenous *nhomie* and *homie*.

**Fig 6:**
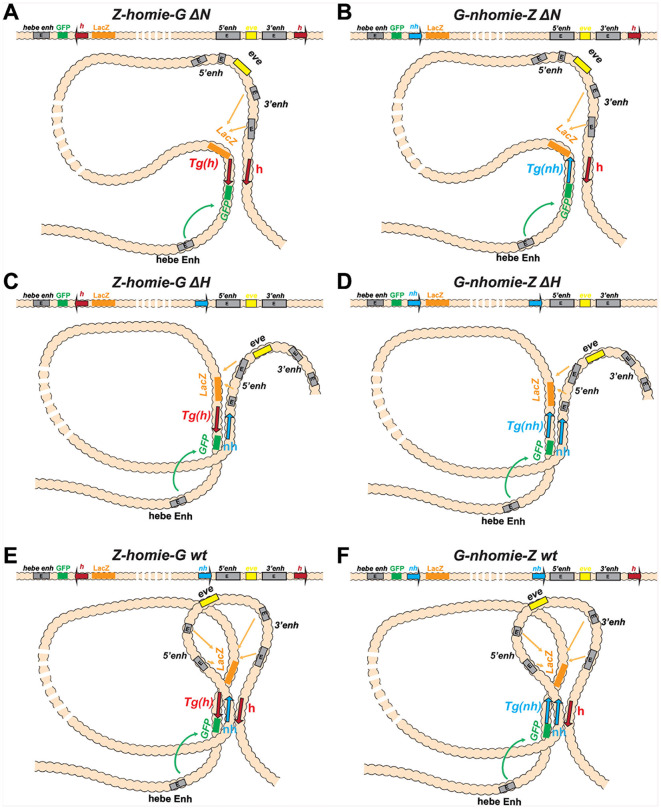
Loop topologies generated by the pairing of transgene and endogenous *eve* insulators. **A.** *Z-homie-G ∆N*: Pairing of the dual reporter containing the *homie* insulator with *homie* on the *∆N* chromosome. Homologous *homie*-*homie* pairing (head-to-head) generates a stem-loop. Since transgene *homie* is oriented in the dual reporter so that it is “downstream” of the *lacZ* reporter, *lacZ* interacts preferentially with the *eve* enhancers. **B.** *G-nhomie-Z ∆N*: Pairing of the dual reporter containing the *nhomie* insulator with *homie* on the *∆N* chromosome. Heterologous *nhomie*-*homie* pairing (head-to-tail) generates a stem-loop. Since transgene *nhomie* is oriented in the dual reporter so that it is “upstream” of the *lacZ* reporter, *lacZ* interacts preferentially with the *eve* enhancers. **C.** *Z-homie-G ∆H*: Pairing of the dual reporter containing *homie* to *nhomie* on the *∆H* chromosome. Heterologous *homie*-*nhomie* pairing (head-to-tail) generates a circle-loop. Since transgene *homie* is oriented in the dual reporter so that it is “downstream” of the *lacZ* reporter, *lacZ* interacts preferentially with the *eve* enhancers. **D.** *G-nhomie-Z ∆H*: Pairing of the dual reporter containing the *nhomie* insulator to *nhomie* on the *∆H* chromosome. Homologous *nhomie*-*nhomie* pairing (head-to-head) generates a circle-loop. Since transgene *nhomie* is oriented in the dual reporter so that it is “upstream” of the *lacZ* reporter, *lacZ* interacts preferentially with the *eve* enhancers. **E.** *Z-homie-G wt*: Pairing of the dual reporter containing *homie* with the wild-type *eve* locus. In the endogenous *eve* locus, heterologous *homie*-*nhomie* pairing (head-to-tail) generates a stem-loop. Transgene *homie* pairs head-to-head with endogenous *homie*, while it pairs head-to-tail with endogenous *nhomie*, generating a complicated multi-loop topology. Since transgene *homie* is oriented in the dual reporter so that it is “downstream” of the *lacZ* reporter, *lacZ* interacts preferentially with the *eve* enhancers. **F.** *G-nhomie-Z wt*: Pairing of the dual reporter containing the *nhomie* insulator to the *eve* locus. In the endogenous *eve* locus, heterologous *homie*-*nhomie* pairing (head-to-tail) generates a stem-loop. Transgene *nhomie* pairs head-to-head with endogenous *nhomie*, while it pairs head-to-tail with endogenous *homie*, generating a complicated multi-loop topology. Since transgene *nhomie* is oriented in the dual reporter so that it is “upstream” of the *lacZ* reporter, *lacZ* interacts preferentially with the *eve* enhancers.

While the loop configuration that is generated by the pairing of the transgene with *nhomie* and *homie* in the wild-type *eve* locus is unclear, the pairing interactions between *homie* and either Z-*homie*-G or G-*nhomie*-Z in *∆N* should generate a stem-loop structure (illustrated in Fig 6A, 6B), while they would generate circle-loop in *∆H* (illustrated in Fig 6C and 6D). In these topologies, sequences within the loop on either side near the paired insulators would be brought into close proximity. Thus, without elements elsewhere in the *eve* locus that can anchor sustained contacts with the transgene, one would expect that, as the distance increases from the paired insulators to the interacting sequences in the transgene and *eve*, the weaker the contacts between them will be. Indeed, this is what is observed in *∆N* (Fig 5A and 5B: 2^nd^ panels; Fig 5C: 2^nd^ and 4^th^ panels). The physical interactions between *lacZ* and *eve* peak at the 3’ end of the *eve* locus (where *homie* is located). The interactions then become progressively weaker, the farther the *eve* sequences are located from the paired insulators (i.e., moving to the left from the peak). We quantitated the contact frequency between *lacZ* and sequences upstream and downstream of the *eve* transcription unit in the wild-type and *∆N* backgrounds. This analysis showed that there is a bias in favor of the enhancers downstream of the *eve* transcription unit (Fig 5E). We also examined the interactions between the *gfp* reporter (which is expressed very weakly relative to *lacZ*) and *eve* (Fig 5D). In wild type, there are *gfp* peaks at *nhomie* and *homie*, while interactions within the *eve* locus are lower than those with *lacZ* as the viewpoint. In *∆N*, interactions are partially retained on the *homie* side, but much reduced over the remainder of the *eve* locus. Additionally, *gfp* contacts sequences beyond the two boundaries of the neighboring TADs more than *lacZ* does, especially toward the 3’ neighboring TAD. This is consistent with the fact that the stem-loop topology brings sequences beyond the paired insulators, which include *gfp*, into closer proximity (illustrated in Fig 6A and 6B).

As noted above, one possible explanation for the increase in *lacZ* expression in the single-insulator deletions is that insulator pairing is strictly pairwise. In this model, the transgene insulator in wild type would have to compete with the pairwise interaction between the endogenous insulators. Since the endogenous insulators are less than 20 kb apart, pairing between them would be favored. In contrast, this competition is removed in the insulator deletions. This could greatly boost pairing between the transgene insulator and the remaining endogenous insulator, if the endogenous insulators are paired most of the time in wild type. So, if insulator pairing were strictly pairwise, there would likely be a significant increase in the contact frequency between the *lacZ* reporter and *eve* in *∆N* compared to wild type. However, the frequency of physical contacts between the *lacZ* reporter and *eve* in *∆N* is not significantly different from wild type (Fig 5F). This would suggest that we need an alternative to the simple pairwise interaction model. In one such model, the topology in wild type, when the transgene is “engaged” with the endogenous locus, involves the formation of a 3-way complex (illustrated in Fig 6E and 6F). In the simplest form of this model, the expression of the *lacZ* reporter in an *eve*-like pattern is facilitated by this single 3-way complex, rather than being a composite of two alternative pairwise complexes (see Discussion for more detail and additional considerations).

### Effects of endogenous insulator removal on *eve* function

**Fig 7:**
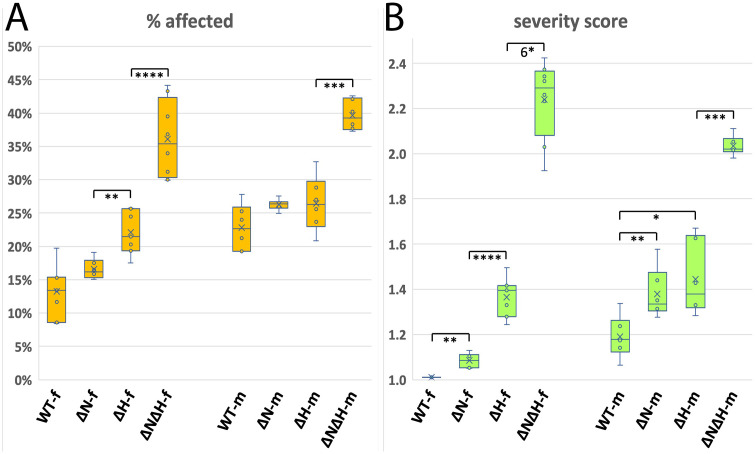
Deleting both *nhomie* and *homie* from endogenous *eve* compromises function more than does deleting only *homie* or *nhomie*, when the genetic background is sensitized to a reduction in *eve* function. *Sco/Cy, ∆N/Cy, ∆H/Cy*, and *∆N∆H/Cy* lines were crossed with *Df(2R)BSC158/Cy*, and embryonic cuticle defects were counted. The parental source of the chromosomes analyzed is indicated as –f (female) or –m (male). The percentage of embryos showing segmentation defects (left graph, % affected) and the average number of ventral abdominal denticle bands deleted per non-wild-type embryo (right graph, severity score) are shown as box-and-whiskers plots. The pair-wise significance of differences (p-values) is indicated as: *: p < .05, **: p < .01, ***: p < .001, ****: p < .0001, 6*: p < 10^–6^. The data are from two separate 3-way comparison experiments, involving [*Sco/Cy, ∆N/Cy,* and *∆H/Cy*] and [*Sco/Cy, ∆H/Cy*, and *∆N∆H/Cy*], and these two data sets were combined as described in Materials and Methods. The number of cuticle preparations used (n) and the total number of embryos counted were as follows: WT-f (n = 7, 1414 embryos), *∆N*-f (n = 6, 1676), *∆H-*f (n = 7, 1885), *∆N∆H*-f (n = 8, 1391), WT-m (n = 6, 1311), *∆N*-m (n = 6, 1676), *∆H*-m (n = 6, 1928), *∆N∆H*-m (n = 6, 2541).

Does removal of an insulator from the *eve* locus affect its function? In a previous study using the *nhomie* deletion *∆N*, we found that homozygous mutants were viable and fertile. However, *∆N* is weakly haploinsufficient, so that when in trans to the *BSC158* deletion, nearly 10% of embryos were missing two or more ventral abdominal denticle bands [25]. To extend this analysis, we have now analyzed embryos derived from self-crossing *∆N∆H*/*SM6a,Cy*, ∆*H/SM6a,Cy*, and homozygous *∆N*, for cuticular segmentation defects. As controls, we used *Sco/SM6a,Cy* (“*Sco/Cy*”), controlling for the *SM6a* chromosome in two of the lines, and *yw*, as the control for the *∆N* self-cross. In all these cases, more than 98% of embryos showed a wild-type larval cuticle phenotype at the end of embryogenesis (S4 Fig). In contrast, embryos from a cross of *Sco/Cy* x *BSC158/Cy* showed a missing ventral abdominal denticle band (most of them in the abdominal segment A6) in ~10–25% of embryos (Fig 7, “WT” in “% affected”). The *Sco/BSC158* and *Cy/BSC158* progeny (which each have a single copy of *eve*) together represent about 50% of these embryos, so this phenotype is not fully penetrant. However, the fact that progeny from the above self-crosses did not show such segmentation defects suggests that homozygotes for the insulator deletions have more than one wild-type copy’s worth of normal *eve* activity (see below and Discussion).

Since we found that *∆N* is weakly haploinsufficient, we crossed each of the three insulator deletion lines with a series of different *eve* mutant chromosomes, as diagrammed in Fig 1B. For *∆nhomie*, we used *∆N*/*SM6a,Cy* in order to make the crosses equivalent, even though the *∆N* lines are homozygous viable and fertile. To compare the number and severity of segmentation defects, we calculated two measures: the percentage of affected embryos in each population (Fig 7A, % affected) and the average number of ventral abdominal denticle bands deleted in the affected population (Fig 7B, severity score). When placed over the *BSC158* chromosome, both *∆H* and *∆N∆H* significantly increased the percentage of embryos showing segmentation defects over that of the control *Sco/Cy* (“WT”, Fig 7A and S1 File) in at least one direction of the cross. In order to present all of the data with this set of crosses together, the data from the experimental set [WT, *∆N,* and *∆H*] (S5A Fig) were merged with those from the experimental set [WT, *∆H*, and *∆N∆H*] to produce Fig 7 (see Materials and Methods). The statistical analysis used the WT data from the latter experiment, which had a larger variance than the WT data from the former experiment. In the former experiment, *∆N* did show a significant increase in the percentage of defects over WT (S5A Fig and S1 File), even though this same comparison in the merged data set did not show a significant difference (Fig 7 and S1 File). Importantly, the severity of defects in each of the 3 cases was significantly increased over that of WT. Furthermore, the percentage of defective embryos and the severity of segmentation defects were both greater with *∆N∆H* than with either *∆N* or *∆H* alone (Fig 7B and S1 File).

Since the *BSC158* deficiency is large and deletes many other genes besides *eve*, we further tested these effects for *∆H* and *∆N∆H* using both *Df(2R)eve* and *eve*^*R13*^ [41]. *Df(2R)eve* deletes most of the *eve* locus plus several kilobases upstream (sequences of junction fragments are shown in S1 Fig), while *eve*^*R13*^ is a point mutant causing premature termination of the Eve protein [32]. The effects of combining these two *eve* mutants with the insulator deletions are similar to those observed for the *BSC158* deficiency (S5 and S6 Figs). This indicates that the *BSC158* results can be attributed to the removal of one functional copy of *eve* in combination with the loss (in trans) of one or both *eve* insulators in the remaining copy, which is only partially functional without the insulators. This interaction is shown in a box plot for *Df(2R)eve* in S5B Fig, and a stacked graph in S6B Fig, while S5C and S6C Figs show the data for *eve*^*R13*^. We also note that in the *Df(2R)eve* crosses, the effect of *∆N∆H* (S5B and S6B Figs, and S1 File) is significantly more severe when it comes from the female parent, for unknown reasons.

In all of the cases described above, virtually all of the missing abdominal denticle bands occurred in even-numbered segments (A2, A4, and A6). This is significant because it is mostly the even-numbered segments that are deleted in *eve* deficiency mutants [33,41]. This indicates that the defects are most likely due to a loss of *eve* function in early embryos.

#### *eve and engrailed* expression are disrupted in *eve* insulator deletion mutants.

The segmentation defects in embryos trans-heterozygous for the insulator deletions and the different *eve* mutants are expected to be due to alterations in the pattern of *eve* expression during the blastoderm stage. S7A Fig shows the pattern of *eve* expression detected by *in situ* hybridization in three representative wild-type, *+ /BSC158*, and *∆N∆H/ BSC158* stage 5 embryos. As expected from the incomplete penetrance of the segmentation defects in *+/BSC158* embryos, *eve* expression in many *+ /BSC158* embryos is indistinguishable from the wild-type control, except for generally weaker expression. However, in a subset of the *+ /BSC158* embryos, expression in stripes 5 and 6 is more severely reduced. The effects on *eve* expression are more pronounced in *∆N∆H/BSC158* embryos, in that *eve* expression is more clearly reduced in all of the stripes, and this reduction is typically greater in stripes 4, 5, 6, and 7.

Embryos from these crosses were then analyzed for *engrailed* (*en*) expression, which is a critical downstream target of *eve* (S7B Fig) [42–45]. We examined the pattern of *en* expression in wild type, *+ /BSC158*, and *∆N∆H/BSC158* at different stages of development. In embryos with the genotype *+ /BSC158*, which have only one copy of endogenous *eve*, *en* expression is abnormal from the time it is initiated. Instead of a relatively even spacing of the 14 *en* stripes, the stripes are “twinned” (S7B Fig, compare wild type and +/*BSC158*). This is due to decreased *eve* expression, which causes a narrowing of the parasegments that span the 7 early *eve* stripes, by a mechanism that has been described in detail [46–50]. Numbers in S7B Fig (stage 7) show the positions of these 7 *eve* stripes relative to the 14 *en* stripes. As a downstream consequence of this narrowing, there is occasional “degeneration” of the parasegment at later stages, resulting in ectopic and/or haphazard *en* expression within the narrowed parasegment (arrowheads in stage 13 embryos). This type of defect is the presumed precursor of the cuticle defects quantified in Figs 7, S5, and S6. An example of an embryo with such defects in 4 adjacent parasegments is shown in S7B Fig, 3^rd^ column (*∆N∆H/BSC*, stage 13), presumably representing an embryo that will develop the severe pattern of defects with 4 denticle bands deleted, a phenotype only very rarely observed in the *+ /BSC158* control.

### Endogenous insulator removal allows the *eve* Polycomb domain to spread

**Fig 8:**
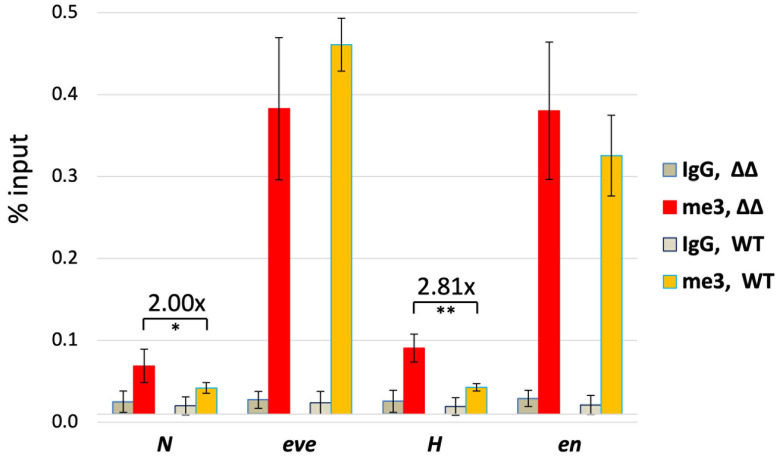
H3K27me3 levels upstream and downstream of *eve* increase when *nhomie* and *homie* are removed. Both *Sco/Cy* (WT) and *∆N∆H/Cy* (∆∆) lines were subjected to ChIP analysis using anti-H3K27me3 (me3) antibody or negative control IgG (IgG). Immunoprecipitated DNA was analyzed by qPCR at 4 positions, as follows. N: upstream of the *nhomie* deletion (*nhomie* is replaced by λ DNA), *eve*: *eve* coding region, *H*: downstream of the *homie* deletion, *en*: the *engrailed* coding region (positive control). The average and standard deviation from 3 independent experiments are shown as % input. The pair-wise significance of differences (p-values) between ∆∆ and WT is indicated as: *: p < .05, **: p < .01. The increase upstream of *nhomie* (∆∆/ WT, after each IgG background is subtracted) is 2.00-fold, while that downstream of *homie* is 2.81-fold, as indicated.

In previous studies, we found that *homie* blocked the spread of the repressive Polycomb histone modification H3K27me3 written by Polycomb Repressive Complex 2 [11]. We also showed that removing *homie* from a modified transgenic *eve* “pseudo-locus” (in which the *eve* coding region is replaced with a *lacZ* reporter gene, and the *TER94* coding region is replaced with a *gfp* reporter) caused the spreading of H3K27me3 into *TER94*, and reduction of *TER94-gfp* transcript levels [51]. Those data suggested that *homie* prevents the spreading of repressive H3K27me3 in order to prevent the repression of *TER94*. Here, we tested whether we could see similar spreading of H3K27me3 outside of the endogenous *eve* locus in the absence of *nhomie* and *homie*, using the *∆N∆H* mutants. It is important to note that this experiment differs from the transgenic experiment in that *∆N∆H* is homozygous in only 1/4 of the embryos from the cross and is heterozygous in another 1/2 of the population, so H3K27me3 is expected to increase outside the *eve* locus in at most 1/2 of the chromosomes being analyzed in the experiment. Despite this difference, which is expected to reduce measured Polycomb spreading by at least 2-fold, we measured a significant increase in H3K27me3 outside the endogenous *eve* locus in both directions (Fig 8: 2.0-fold increase upstream of *nhomie*, 2.8-fold increase downstream of *homie*) with the removal of both *nhomie* and *homie*. In the previous experiments using the *eve* pseudo-locus [51], we found that removing *homie* (along with the 3’ PRE) resulted in a 5.7-fold increase in H3K27me3 levels just downstream. Since in the present study, less than half of the chromosomes in the analyzed population have *homie* (along with the PRE) deleted, whereas in that study all the chromosomes had the deletion, this compares very well to the 2.8-fold change seen here. So, while these increases are not dramatic, they may be significant for the proper expression of neighboring genes.

## Discussion

### Insulators function to isolate regulatory domains

Insulators function to prevent enhancers or silencers in one TAD from influencing genes in neighboring TADs. Our analysis of the phenotypic consequences of the *homie* and *nhomie* deletions provides strong support for this hypothesis. In Ke, et al. [25], we showed that the *eif3j* gene, which is located upstream of the *nhomie* insulator, is inappropriately activated by *eve* enhancers when *nhomie* is deleted. In addition to restricting the activity of enhancers, another key function of the *eve* insulators in their endogenous setting is to “contain” repressive chromatin within the *eve* domain, presumably thereby facilitating the full levels of expression of neighboring transcription units. We previously showed that *homie* has this function in the context of an *eve* “pseudo-locus” transgene, where a modest increase in H3K27me3 levels was sufficient to significantly reduce *TER94* promoter activity [51]. Consistent with that study, there is a similar spreading of the *eve* Polycomb domain in both directions in the absence of the endogenous *eve* insulators, measured here as an increase in the characteristic histone modification written by Polycomb Repressive Complex 2, H3K27me3 (Fig 8).

One of the key activities of insulators is the establishment of units of independent gene activity [6,52–54]. The studies here also support a related function, that insulators help to facilitate full levels of expression of “insulated” transcription units. For *eve*, this manifests itself in both the single and the double insulator deletions as an increase in embryonic defects. These defects are characteristic of reduced *eve* function at a critical early stage (Figs 7, S5, and S6), when pattern formation is highly dependent on properly formed “pair-rule” *eve* stripes [33,42–45,55]. The establishment of fully functional parasegment spacing is known to be highly sensitive to the levels of *eve* function [46,47] relative to that of its “complementary” pair-rule gene *fushi tarazu* (*ftz*) [45,56]. Specifically, the positioning of the 14 stripes of the segment polarity gene *engrailed* (*en*), which is immediately downstream of *eve* and *ftz* in the segmentation hierarchy [46,47], become abnormally spaced when *eve* function is reduced [42–46] or when *ftz* function is increased [57]. In both cases, each odd-numbered *en* stripe becomes closer to the next (posteriorly adjacent) even-numbered *en* stripe. This effect occurs in heterozygotes for *eve* null mutations, yet this abnormal spacing is almost always corrected as pattern formation continues, and few abnormal embryos are seen at the end of embryogenesis (Figs 7, S5, and S6). However, when *eve* function is reduced further, a critical point is reached where the narrow spacing is no longer efficiently corrected, and defects increase significantly. This is seen as “skips” in the pattern of denticles within even-numbered abdominal segments (this is the phenotype for which *eve* is named, which was based on the initially isolated, strong hypomorphic allele *eve*^*1*^) [33,55]. The results here show that *homie* deletion causes segmentation defects in a sensitized background with one copy of endogenous *eve* function. Deletion of *nhomie* in addition to *homie* increases the severity of the phenotype, indicating that proper activation of *eve*, mediated by efficient interaction between *eve* enhancers and the promoter, requires both *homie* and *nhomie*. We note that because our *homie* deletion is imprecise*,* the extra deleted sequence could contribute to its more severe phenotype relative to deleting *nhomie* alone. For example, the deletion of the *eve* 3’ PRE might reduce the stability of *eve* enhancer-promoter interactions. Nonetheless, our results indicate that the *eve* insulators, both together and individually, contribute to establishing the proper level of expression and function of the *eve* locus. While interfering with each of these three functions of the *eve* insulators, namely facilitating full *eve* activity, preventing the spread of repressive chromatin, and restricting the activity of *eve* enhancers, produce incompletely penetrant defects on their own, they are probably a sufficient driving force for the evolution of a well-insulated *eve* TAD. Similarly subtle yet important functions of insulators are likely to apply genome-wide.

Consistent with our results with endogenous *homie* and *nhomie*, a recent study showed that LR enhancer-promoter interactions were increased by flanking a transgene with *homie* and *nhomie* [58]. How do the *eve* insulators upregulate transcription? Loss of TAD boundaries could cause more frequent accessing of neighboring promoters by *eve* enhancers. Where *eve* enhancer activity is limiting, these interactions could reduce *eve* transcriptional output by promoter competition. Consistent with this idea, Chen et al. [27] found that the presence of a *homie*-containing reporter transgene at -142 kb, similar to those used in this study, induced a weak loss-of-function phenotype, much like the insulator deletions described here.

### Competition between cis and trans insulator interactions

We observed that transgenic reporter expression is stronger in late-stage embryos when a trans copy of the *eve* locus is absent (due to the chromosomal deficiency *BSC158*). However, when the *∆N∆H* chromosome was in trans, the expression was not increased (Fig 4), consistent with a lack of competition. Therefore, this effect is probably not due to competition between the trans copy of the endogenous insulators and the pairing of the transgene with the cis copy of the endogenous insulators. However, it should be noted that trans pairing has been suggested to be weaker than cis pairing [59], potentially explaining this result without dismissing the importance of pairwise competition among insulators.

A more likely explanation for the effect with *Df(2R)BSC158* is that with a large chromosomal deficiency, homologous chromosomes are no longer paired in the “missing” region, which here contains the *eve* locus plus about 100 kb upstream of *eve* (and ends about 40 kb downstream of the transgene insertion site; see Fig 1 legend for precise locations). This may lead to a greater flexibility of the chromatin fiber for most of the region between the *eve* locus and the transgene, increasing the frequency with which they find each other by random flexing of the chromosome. This flexibility could thus lead to a more efficient LR cis interaction [60], and increase the number of cells that express the transgene reporter in an *eve* pattern. Furthermore, the wild-type chromosome is expected to pair with the *BSC158* deficiency chromosome both upstream and downstream of the missing region, creating a “loop out” of the hemizygous region (which contains the *eve* locus). The transgene insertion site, which is upstream of the missing region (and so is presumably in a mostly paired region) is thus brought closer to the region downstream of *eve*, beyond the missing region. This would then bring the transgene closer to the *eve* locus in 3-D space, likely increasing their frequency of interaction. Such a mechanism would be consistent with our earlier observation of distance-dependent transgene – endogenous *eve* interactions [24].

### Pairwise interactions between transgenic and endogenous *eve* insulators change both transgene reporter expression and its physical interactions

As would be expected from the known properties of Drosophila insulators, the LR regulatory interactions seen between *homie-* and *nhomie*-containing transgenes and the *eve* enhancers require endogenous *homie* and *nhomie* (Fig 2). On the other hand, LR trans pairing by transgenic copies of either *homie* or *nhomie* can occur when *homie* and *nhomie* are both absent in *cis*, but present in *trans* (Fig 2). Our studies also examine how pairing interactions change depending on the number and position of potential pairing partners. When a transgenic insulator has a choice of either of the two endogenous *eve* insulators as a partner (in the wild-type background), the results are distinctly different than when only one endogenous *eve* insulator is present in the chromosome (in the *∆N* or *∆H* background) (Fig 3). In the latter case, some enhancer-promoter interactions are weakened, consistent with the loss of an interaction with one end of the *eve* locus, while others are strengthened: specifically, those that involve enhancers that are closer to the remaining insulator. This strengthening suggests that the remaining insulator may form a more stable or a more frequently forming complex when the other endogenous insulator is missing. This, in turn, is suggestive of a competition occurring in wild-type between the two endogenous insulators for the transgenic copy.

There are other likely contributing factors for the observed changes in *eve* enhancer interactions with a transgenic promoter when one endogenous insulator is missing. Assuming that transgenic and endogenous promoters are competing for enhancer activity, then anything that reduces endogenous promoter access is expected to increase transgene reporter expression (in this case, *lacZ*). We showed here that removing either *homie* or *nhomie* from the *eve* locus reduces *eve* function at early stages, and this likely occurs through a small decrease in expression in early stripes. This may allow enhancers to access the *lacZ* promoter more efficiently and increase reporter gene expression. Additionally, Micro-C data showed that the endogenous *homie* region interacts with the endogenous promoter, as well as with the *nhomie* region [25], and disruption of these interactions by deleting either *homie* or *nhomie* may well reduce the efficiency of endogenous enhancer-promoter interactions, allowing greater expression of the transgenic promoter. Furthermore, the endogenous *homie-nhomie* interaction loop can no longer form on either the *∆N* or the *∆H* chromosome. This may reduce endogenous *eve* promoter access to the more distant enhancers, as they are further away, on average, than they are when the two endogenous insulators are paired. This, in turn, can reduce competition between the *eve* and transgene promoters, allowing greater reporter gene expression.

Interestingly, beyond the distinct bias in reporter gene activity driven by either the upstream or downstream *eve* enhancers when the transgene insulator pairs with the remaining endogenous insulator in *∆H* and *∆N*, we did not see evidence that activation of *lacZ* depends on distance of the individual enhancers from the tethering point (which is the remaining endogenous insulator). For example in *∆N*, stripes 4 and 6 are expressed as strongly as is stripe 5, although the position of the stripe 4 + 6 enhancer is considerably further away from endogenous *homie* than is the stripe 5 enhancer. Rather, the upstream and downstream enhancers of *eve* seem to behave as two separate units in activating the transgene reporter. This could be due to the upstream *eve* promoter region acting as a weak insulator [61]. In fact, an interaction between *homie* and this region is seen in MicroC data [25], as mentioned above.

### Pairwise versus tripartite interactions

As described above, when one of the *eve* insulators is deleted, there is not only a change in the pattern of regulatory interactions between the *lacZ* reporter and the *eve* enhancers, there also appears to be an increase in the level of *lacZ* expression within the stripes that are preferentially activated (Fig 3). One possible explanation for such increased expression is that insulator pairing interactions are strictly pairwise. In this case, in a wild-type background, there would be a competition between the transgene insulator and the two *eve* insulators. Since proximity would favor pairing of the *eve* insulators with each other at the *eve* locus, the frequency of pairing interactions between the transgene insulator and either one of the *eve* insulators would be suppressed by competition. That is, assuming endogenous *homie* and *nhomie* are paired with each other most of the time, they would need to disassociate before either could pair with the transgene insulator, and this would suppress pairing interactions with the transgene insulator. So, because in the *∆H* and *∆N* deletions the competing endogenous insulator is removed, this would be expected to increase the frequency of insulator pairing between the transgene and the remaining *eve* insulator. Consistent with this possibility, the level of *lacZ* expression from the Z-*homie*-G and G-*nhomie*-Z transgenes is noticeably higher in the *∆N* deletion, and to a lesser extent in *∆H*, than in wild type (Fig 3). However, the MicroC data challenge this explanation. In this case, when one of them is deleted, we would expect the transgene insulator interactions with the *eve* locus to increase significantly. Contrasting with this prediction, there is no significant change in total transgene – *eve* locus interactions when one endogenous insulator (*nhomie*) is deleted (Fig 5F). Therefore, the MicroC results are incompatible with this simple model based on strictly pairwise competition.

One possible way to reconcile the MicroC data with a pairwise-only interaction model is to include homolog interactions in the model. When homologs are paired, there are two copies of endogenous *homie* present near each other, even when *nhomie* is deleted. If, instead of local cis interactions (since *nhomie* and *homie* no longer pair in cis), *homie-homie* interactions in trans are present most of the time in most cells when *nhomie* is deleted (in *∆N*), then the competition barrier to transgene interactions with endogenous *eve* might not be reduced very much. Thus, we might expect the total amount of transgene – endogenous *eve* interactions to remain about the same when *nhomie* is deleted, consistent with what is seen (Fig 5F). Arguing against this model is that homolog pairing between enhancers and promoters has been shown to be considerably weaker than local cis pairing, at least in some parts of the genome [59].

Another way to explain the MicroC results is to modify the simple pairwise-only interaction model by including a multi-component complex in the mix. In the simplest form of this model, a tripartite complex can form, involving simultaneous endogenous *nhomie*-*homie* interactions with binding by the transgene insulator. In this model, the pre-existence of the local *nhomie-homie* complex does not present a barrier to transgene interactions with the *eve* locus, and so no increase in physical interactions is expected when one endogenous insulator is deleted (this situation is illustrated in Fig 6E and 6F).

A hybrid model can also potentially reconcile transgene expression with the MicroC data. In the simplest version of this model, the interaction that initially “captures” the distant transgene is a tripartite complex like that described above. This complex, involving the two endogenous *eve* insulators and the transgene insulator, holds the transgene in proximity to the *eve* locus and mediates most of the physical contacts seen by MicroC. This is plausible because a pairwise complex between two “matched” insulators like *homie* and *nhomie* likely involves a number of individual pairwise contacts between insulator proteins arrayed along their length. At any given time, some of those contacts may not be engaged, and could therefore present an interaction interface. These unengaged contact points would be free to interact with a 3^rd^ compatible partner to form a tripartite complex. This configuration could itself be quite stable, particularly if individual insulator-bound proteins with multiple interactions domains can interact simultaneously with proteins bound to more than one other insulator. Furthermore, it would be expected to “isomerize” at some frequency to either of 3 pairwise complexes, only one of which would disengage the attachment of the transgene to the endogenous locus. The remaining two, namely the transgene insulator paired with either *nhomie* or *homie*, might be more conducive to *lacZ* expression driven by the nearby *eve* enhancers than is the tripartite complex. In this more dynamic, hybrid model, expression of *lacZ* comes more from the pairwise complexes, while the MicroC signal is a composite of all the alternative complexes, most of which may be tripartite. This would naturally explain both the “extra” enhancement of *lacZ* expression when one endogenous insulator is removed (particularly in stripes 4, 5, and 6 in the *∆N* combinations), and the lack of a corresponding increase in MicroC signal in *∆N* over wild type. This type of model is also compatible with the results of insulator competition experiments [62–64], which were analyzed based on reporter gene expression only, before chromosome conformation capture methods were developed. These results were consistent with pairwise complexes being primarily responsible for the observed expression patterns. Why might pairwise complexes give more reporter expression? The tripartite complex could be more constrained, while a pairwise complex might be more flexible, allowing enhancer-promoter interactions to form more efficiently or to be more stable.

While the MicroC experiments suggest that insulator pairing interactions may not be exclusively pairwise, further studies are required to disentangle the properties of tripartite and pairwise interactions. Since insulator pairing depends on protein-protein interactions, the combinations that are possible may be different when insulator interactions are pairwise versus when they are tripartite. Given the increase in stripe expression in stage 5 embryos that is observed when one endogenous insulator is removed, it seems possible that pairwise complexes between insulators are formed more rapidly than tripartite complexes. As the cell cycle in most of the cells in late-stage embryos is lengthy compared to those in pre-cellular blastoderm embryos, there would be much more time to establish tripartite complexes. The situation is also different in stage 5 embryos in that the contacts between the transgene insulator and *eve* insulators have to be established *de novo* [27]. It would certainly be interesting to follow the dynamics of pairwise versus tripartite pairing interactions in early embryos using live imaging.

## Materials and methods

### Creation of *nhomie* and *homie* deletions, and breakpoint analysis of chromosomal deficiencies

Creation of the *∆N* line has been described previously [25]. In short, we inserted two attP sites flanking endogenous *nhomie*. To do this, we used a donor plasmid, P-attPx2-*mw,* carrying two 102 bp attP sequences flanking a modified *mini-white* (*mw*) gene. The following modification was made to *mw*: the Wari insulator [65] was deleted from the standard *mw,* and Glass binding sites were added to boost the eye color. The endogenous *nhomie* region was replaced with this attP-*mw*-attP using CRISPR. This chromosomal modification resulted in one attP site being inserted in the intron of *CG12134*, and the other being inserted between the *eve* late-stripe enhancer and the 3 + 7 stripe enhancer (Fig 1C). This process also deleted 2.2kb of endogenous sequence, including *nhomie* and the *eve* late-stripe enhancer. After identifying a successful insertion (*NattPmw*), *mw* was replaced by the same 2.2kb sequence, but with 600 bp of phage λ DNA in place of 600 bp *nhomie*, using recombinase-mediated cassette exchange (RMCE) [66].

Several attempts to insert attP sites flanking *homie* by CRISPR [30,31] using different gRNA sets failed. While we do not fully understand why our multiple trials of this strategy did not work, it is possible that removing *homie* is either dominantly lethal, or that it reduces viability such that recovery of the resulting chromosome is difficult, perhaps due to the function of *homie* in preventing repression of the adjacent, essential gene, *TER94*. However, we have a homed transgenic line that inserted at +9111 bp relative to the *eve* transcription start site [24]. Using this line, we successfully mobilized the transgene, creating a small deletion (2R:9987986–2R:9989354 fusion, the two ends share the sequence AAA) (Fig 1B, sequence is shown in S1 Fig). The deletion includes the *eve* 3’ PRE, *homie*, and the first exon of *TER94* (Fig 1B). We described this line as *∆H*. *∆H* is homozygous lethal*.* The same strategy that was used to make the *nhomie* deletion on a wild-type chromosome was used to modify *nhomie* on the *∆H* chromosome, creating the *∆N∆H* deficiency chromosome (Fig 1C). As expected, *∆N∆H* is homozygous lethal. Lines carrying either the *∆H* or *∆N∆H* chromosome over a balancer were self-crossed to make *∆H* or *∆N∆H*, respectively, (“*∆∆*” in some figure labels) homozygous embryos.

For *Df(2R)eve* [33,41], information in Flybase [39] suggested that its breakpoints are in *Mef-2* and *TER94*, so we tested potential PCR primers to find ones that amplified junction fragments. These PCR products were then sequenced to identify the specific breakpoints (S1 Fig). The PCR primers used to amplify the breakpoint junction fragments are: for the *∆H* deletion, CAGTCGAGCCTCCGTAAGGG and CCTCCAGCAAAGGATGACTTG, and for *Df(2R)eve*, TTTCAACCGCACACAATCC and CATTCATTCCAAATCACGCAC.

### Creation of attP sites at –142kb and insertion of reporter transgenes

Two attP sites were inserted near the original -142kb attP site [17,24] on the wild-type, *∆N*, *∆H*, and *∆N∆H* chromosomes, using the same CRISPR strategy described above. Then, *mini-white* was replaced with each of the reporter transgenes using RMCE. Dual reporter transgenes were used (Fig 1D) [17], carrying either 400 bp *homie* (*Z-homie-G*), 600 bp *nhomie* (*G-nhomie-Z*), or 500 bp of *lambda* DNA (*Z-lambda-G*). For each of these, the orientation in which the *lacZ* reporter is more strongly activated due to orientation-specific LR pairing was used in this study.

### Analysis of transgenic lines

*In situ* hybridization was performed based on previously published methods [67], except that RNA was visualized using a histochemical reaction, as described previously [11]. Briefly, approximately 60μl embryos per sample (stage 4–15 embryos were present in each sample) were subjected to the process. DIG-labeled antisense RNA probes against *lacZ*, *gfp, eve, or en* were visualized using alkaline phosphatase-conjugated anti-DIG antibody (Roche), using CBIP and NBT as substrates (Roche). Each set of experiments was carried out with the positive control and experimental samples in parallel to minimize experimental variation. Once color became visible in the positive control, the reactions of all the samples were stopped simultaneously. Each experiment was performed at least twice, with independent *in situ* hybridization procedures. Representative expression is shown in the figures.

### MicroC library construction

Details of the MicroC procedure were described previously [25]. Micro-C libraries were prepared as previously described [68] with the following modifications: 50uL of 12–16hr embryos were used for each biological replicate. 60U of MNase were used for each reaction to digest chromatin to a mononucleosome:dinucleosome ratio of 4. Libraries were barcoded, pooled, and subjected to paired-end sequencing on an Illumina Novaseq S1 100 nt Flowcell (read length 50 bases per mate, 6-base index read).

### Micro-C data processing

MicroC data for *D. melanogaster* were aligned to custom genomes edited from the Berkeley Drosophila Genome Project (BDGP) Release 6 reference assembly [69] with BWA-MEM [70] using parameters **-S -P -5 -M**. Briefly, the custom genomes are created by inserting the transgenic sequence into the –142kb integration site, as predicted from perfect integration. The resultant BAM files were parsed, sorted, de-duplicated, filtered, and split with Pairtools (https://github.com/mirnylab/pairtools). Pairs were removed where only half of the pair could be mapped, or where the MAPQ score was less than three. The resultant files were indexed with Pairix (https://github.com/4dn-dcic/pairix). The files from replicates were merged with Pairtools before generating 100 bp contact matrices using Cooler [71]. Finally, balancing and Mcool file generation was performed with Cooler’s Zoomify tool.

Virtual 4C profiles were extracted from individual replicates using FAN-C [72] at 400 bp resolution. The values were summed across replicates and smoothed across three bins (1.2kb). Viewpoints were determined based on the most informative region for interpretation. We used an 800 bp window in the gene body of either *lacZ* or *gfp*. The raw and processed sequencing data are available under NCBI GEO accession number GSE328676.

### Quantification of total contacts between the *lacZ* reporter and the *eve* locus in wild type and *∆N*

For *G-nhomie-Z* and *Z-homie-G*, the locations of the *lacZ* reporter and the *eve* locus in wild type and *∆N* were identified. Then the interaction region between *lacZ* and the *eve* locus was visualized, in order to manually segment bounding boxes of interaction between them. After matrix balancing and masking of null values, the mean of the contact frequency within this bounding box was taken for each dataset. This became the average contact frequency between the transgene *lacZ* and *eve.*

### Analysis of embryonic cuticle defects

To identify segmentation defects in developing embryos, embryos were collected and analyzed as described previously [25], with minor modifications. Briefly, embryos were collected for 2.5-3 hr, allowed to develop for 20–21 hr at 25°C, then dechorionated and mounted in a 1:1 mixture of Hoyer’s medium and lactic acid. Mounted embryos were left at room temperature until they cleared, and the patterns of ventral abdominal denticles were examined and tallied as follows. Loss of at least one-fifth of a denticle band in A1-A8 was counted as ‘missing’. Fused denticle bands, which rarely occurred, were also counted as a ‘missing’ band. Minor defects such as those within individual denticle rows were not counted.

Wild-type (WT), *∆N, Sco/SM6a,Cy, ∆H/SM6a,Cy*, and *∆N∆H/SM6a,Cy* lines were self-crossed for S4 Fig. For each cross, 3–4 independent cuticle preparations were analyzed. To sensitize for functional differences of these chromosomes, each of the lines was crossed with each of the *eve* heterozygous mutant lines *Df(2R)BSC158/CyO* (Figs 7, S5A, and S6A), *Df(2R)eve/CyO* (S5B and S6B Figs), and *eve*^*R13*^*/CyO* (S5C and S6C Figs). The two directions of each cross were analyzed separately. For each cross, 5–8 independent cuticle preparations were analyzed.

In the sensitized condition, segmentation defects were quantified in two ways: the percentage of embryos with segmentation defects (% affected) and the average number of ventral abdominal denticle bands deleted per non-wild-type embryo (severity score). The data from each set of cuticle preparations are presented as box-and-whiskers plots (Figs 7 and S5), and the pair-wise significance of differences (p-values) between lines was calculated using the t-test function (2-tailed, unequal variances) in Excel (Microsoft). For Fig 7, two sets of data were combined, one directly comparing WT, *∆N*, and *∆H* (shown in S5A Fig) and the other directly comparing WT, *∆H*, and *∆N∆H*. The raw data from this 2nd comparison were used in Fig 7, along with scaled data for *∆N* from the 1st comparison. For this purpose, individual data points from the 1st comparison were scaled based on their values relative to WT and *∆H* (they were all between the average values for WT and *∆H* in the 1st comparison, and were scaled to have the same fractional distance from the average WT and *∆H* values in the 2nd comparison as they did in the first comparison). The statistical analysis for Fig 7 used these scaled values for *∆N* in combination with the raw data for the other lines. For a different visual comparison, stacked graphs are shown in S6 Fig. For this, the percentages of embryos (average and standard deviation) with 4 different severities of phenotype are shown: wild-type, mild (1 band missing), moderate (2 bands missing), and severe (3–4 bands missing). The complete set of statistical calculations is shown in S1 File.

### Analysis of H3K27me3 levels

ChIP-qPCR was performed based on previously published methods [73]. Briefly, stage 4–17 embryos (approximately 200ul) from *Sco/Cy* and *∆N∆H/Cy* were cross-linked in 2% formaldehyde for 10 min. After sonication, 50μg of isolated chromatin was used to immunoprecipitate with anti-H3K27me3 (Millipore), with rabbit IgG (Jackson ImmunoResearch) used as the negative control. Precipitated chromatin samples were collected using ProteinG magnetic beads. After reversing cross-links, purified samples were dissolved in 50 μl TE, and 1 μl was used for each qPCR reaction. Triplicate samples were analyzed by real-time PCR (Life Technologies, StepOne Plus), using SYBR Green Master Mix with ROX dye (Roche Applied Science). Data were analyzed with StepOne software (Life Technologies), using the standard curve method. The data are presented as average % inputs from 3 independent ChIP analyses. Standard deviations were calculated using Excel software (Microsoft). The pair-wise significance of differences (p-values) between lines was calculated using the t-test function (1-tailed, unequal variances) in Excel (Microsoft).

Primers used for qPCR were as follows. *N* (upstream of *nhomie*): CGGAGAATCCGGCATTGTTA and GCTTGCGTGATTTCTTCTCC, *eve* (*eve* coding region): TCCAGTCCGGATAACTCCTTG AAC and TGTAGAACTCCTTCTCCAAGCGAC, *H* (downstream of *homie*): AAGGGCCACATCGCAGACATACTA and GTCGCGGTAAATGTCTTTGTCTCG, *en* (*engrailed* coding region): GAGAACCAGGCCAGCATATT and CTAAACTCCAGCAGATCCACTC.

## Supporting information

S1 Fig Junction sequences of the chromosomal deletions *∆H* and *Df(2R)eve.* Sequences upstream of each junction are shown in boldface. The underlined AAA sequence in *∆H* could be from either side of the junction, since it is found on both sides.
(TIF)

S2 Fig λ DNA does not promote long-range pairing, either with or without the endogenous *eve* insulators. *lacZ* expression from the reporter gene *Z-lambda-G* on wt, *∆N*, *∆H*, and *∆N∆H* chromosomes, labeled as in Fig 2. Note that there is no *eve*-like expression. Consistent with previous studies [11,17], non-*eve* like expression is seen in the form of head stripes (yellow with red outline) and *hebe*-like ventral mid-line expression (blue with red outline). Scale bar: 50μm.
(TIF)

S3 Fig Transgenes behave similarly at an attP site near the original –142kb site. *lacZ* and *gfp* expression from *Z-homie-G* and *G-nhomie-Z*, labeled as in Fig 2. Consistent with previous studies [17, 26], LR pairing is biased toward one transgenic reporter or the other, depending on the orientation of *homie* or *nhomie* in the transgene (see main text). Scale bar: 50μm.
(TIF)

S4 Fig Embryos homozygous for the *homie* deletion and for the *homie*+*nhomie* deletions show few cuticle defects. Lines of *wt, ∆N, Sco/Cy, ∆H/Cy,* and *∆N∆H/Cy* were self-crossed, and cuticle defects were counted as either WT (no missing denticle bands), or as having 1, 2, 3, or more missing denticle bands. For *∆H* and *∆N∆H*, homozygotes are expected to be 25% of the population. Total numbers of counted embryos are shown at the bottom. Number of cuticle preparations included: WT and *∆N*: n = 4; *Sco/Cy*, *∆H/Cy*, and *∆N∆H/Cy*: n = 3.
(TIF)

S5 Fig Deleting either *nhomie* or *homie* from the endogenous *eve* locus compromises embryonic function. Embryonic cuticle defects were tabulated from the following crosses: A. *Sco/Cy* (WT)*, ∆N/Cy* (∆N), and *∆H/Cy* (∆H) crossed with *Df(2R)BSC158/Cy*. B. *Sco/Cy* (WT)*, ∆H/Cy* (∆H), and *∆N∆H/Cy* (∆N∆H) crossed with *Df(2R)eve/Cy.* C. *Sco/Cy* (WT)*, ∆H/Cy* (∆H), and *∆N∆H/Cy* (∆N∆H) crossed with *eve*^*R13*^*/Cy.* The parental source of the chromosome analyzed is indicated as -f (female) or -m (male) after each genotype. The percentage of embryos showing deleted ventral abdominal denticle bands (left graph, % affected) and the average number of denticle bands deleted per non-wild-type embryo (right graph, severity score) are each shown as a box-and-whiskers plot. The pair-wise significances of differences (p-values) are shown as the number of asterisks. *: p < 0.05, **: p < 0.01, ***: p < 0.001, ****: p < 0.0001, *****: p < 10^–5^, *^6^: p < 10^–6^. The number of cuticle preparations included (n) and the total number of embryos counted were as follows: in A, WT-f (n = 6, 2722 embryos), ∆N-f (n = 6, 1676), ∆H-f (n = 6, 3661), WT-m (n = 6, 1119), ∆N-m (n = 6, 1623), ∆H-m (n = 6, 1000); in B, WT-f (n = 6, 2457), ∆H-f (n = 6, 3773), ∆N∆H-f (n = 6, 2624), WT-m (n = 5, 2123), ∆H-m (n = 5, 2320), ∆N∆H-m (n = 5, 2217); in C, WT-f (n = 6, 1457), ∆H-f (n = 6, 3434), ∆N∆H-f (n = 6, 1500), WT-m (n = 5, 2641), ∆H-m (n = 5, 2939), ∆N∆H-m (n = 5, 3289).
(TIF)

S6 Fig Deleting both *nhomie* and *homie* from endogenous *eve* compromises function more than does deleting only *homie.* *Sco* (wild type control from *Sco/Cy* stock)*, ∆H/Cy* (*∆H*), and *∆N∆H/Cy* (*∆N∆H*) were crossed with either, in A, *Df(2R)BSC158/Cy*, or in B, *Df(2R)eve/Cy*, or in C, *eve*^*R13*^*/Cy.* The same data used for Figs 5, S5B, and S5C are shown here as stacked graphs. WT: no missing ventral denticle bands; mild: 1 missing; moderate: 2 missing; severe: 3–4 missing. Tables at the bottom show the average percentages of embryos in each deficiency class (%) and their standard deviations (stdev). Scale bar: 50μm.
(TIF)

S7 Fig Abnormal *eve* and *engrailed* expression is seen in *∆N∆H/Df(2R)BSC158* embryos. A. *eve* expression at stage 5 (early to later stage 5 embryos are shown from top to bottom). B. *engrailed* expression at stages 6, 7, 9, 11, and 13. At stage 7, the positions of early *eve* stripes are numbered. Fused *engrailed* stripes that likely prefigure missing denticle bands are marked with red arrowheads at stage 13. wt: homozygous wild-type genotype (both copies of the *eve* locus are intact), + */BSC*: wild type over *Df(2R)BSC158* (1 copy of the wild-type *eve* locus present), *∆N∆H/BSC*: *∆N∆H* over *Df(2R)BSC158* (1 copy of the *∆N∆H eve* locus present). Scale bar: 50μm.
(TIF)

S1 File Statistical comparisons for Figs 7 and S5. The complete set of pair-wise significance of differences (p-values) between lines, calculated using the t-test function in Microsoft Excel (options settings: 2-tailed, unequal variances). Font colors: red: p > 0.05; black: p < 0.05.
(XLSX)
