# Supplementary figures and images for: Chromatin insulators homie and nhomie can interact with distant copies either together or separately, with distinct outcomes for enhancer-promoter interactions
Source: PLoS Genet. 2026 Jun 5;22(6):e1011940. doi: 10.1371/journal.pgen.1011940 (PMC13240896; doi:10.1371/journal.pgen.1011940)

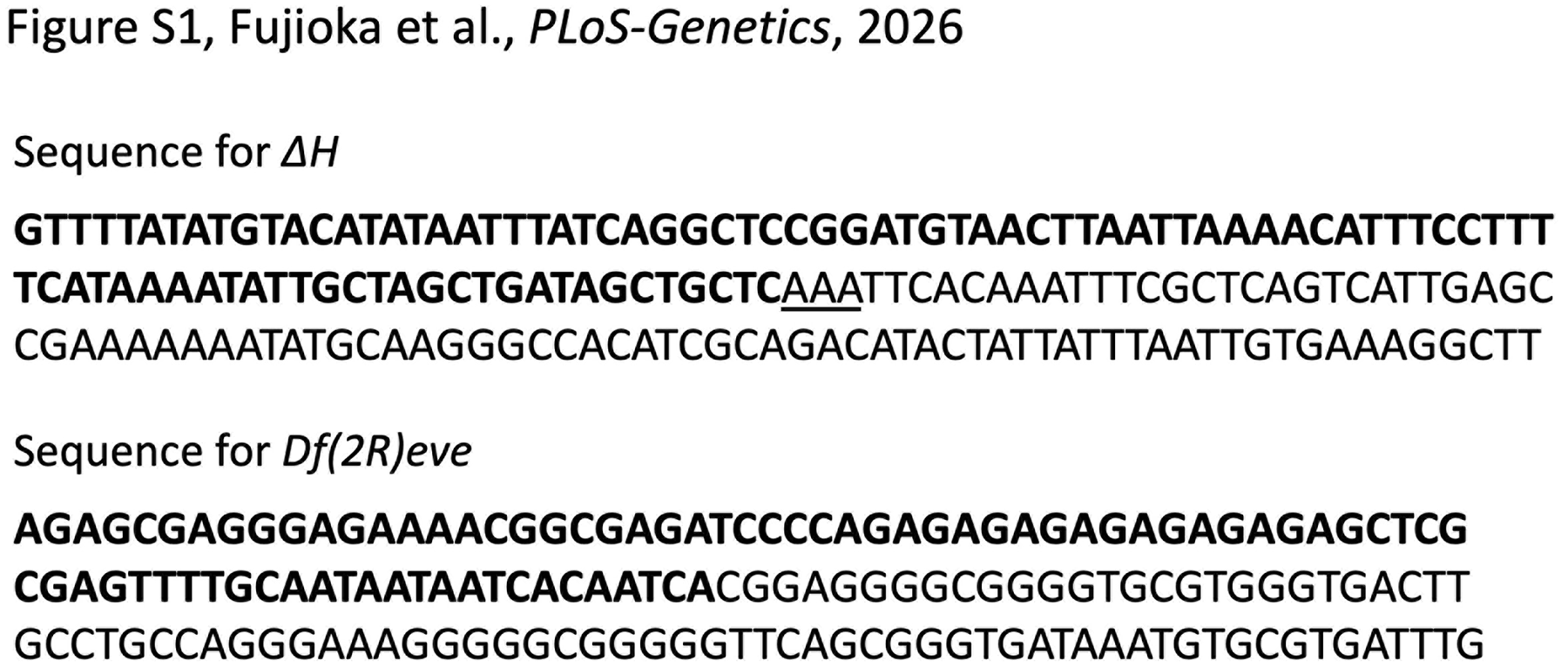

Supplement: S1 Fig — Sequences upstream of each junction are shown in boldface. The underlined AAA sequence in ∆H could be from either side of the junction, since it is found on both sides. (TIF) [file pgen.1011940.s001.tif]

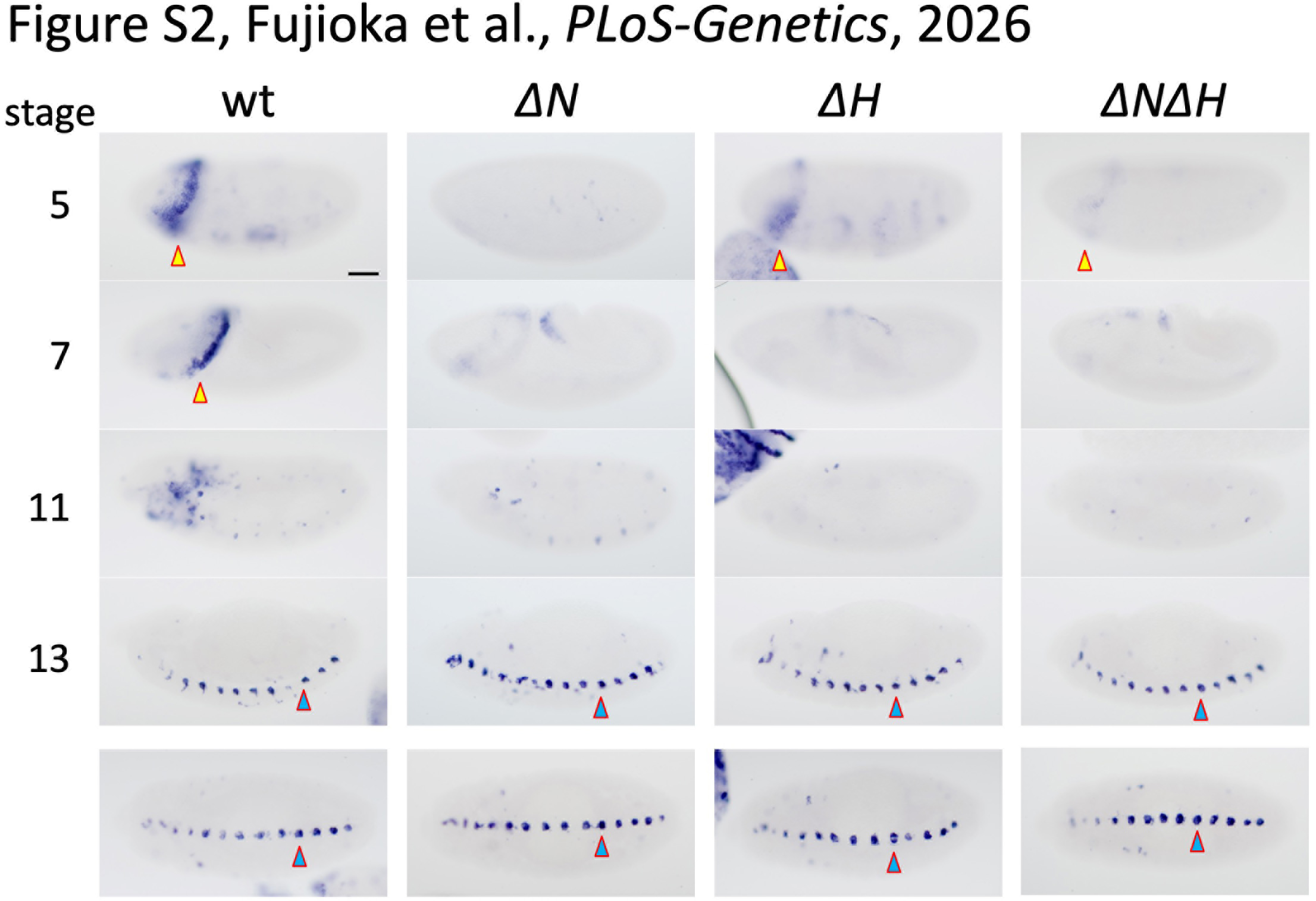

Supplement: S2 Fig — lacZ expression from the reporter gene Z-lambda-G on wt, ∆N, ∆H, and ∆N∆H chromosomes, labeled as in Fig 2. Note that there is no eve-like expression. Consistent with previous studies [11,17], non-eve like expression is seen in the form of head stripes (yellow with red outline) and hebe-like ventral mid-line expression (blue with red outline). Scale bar: 50μm. (TIF) [file pgen.1011940.s002.tif]

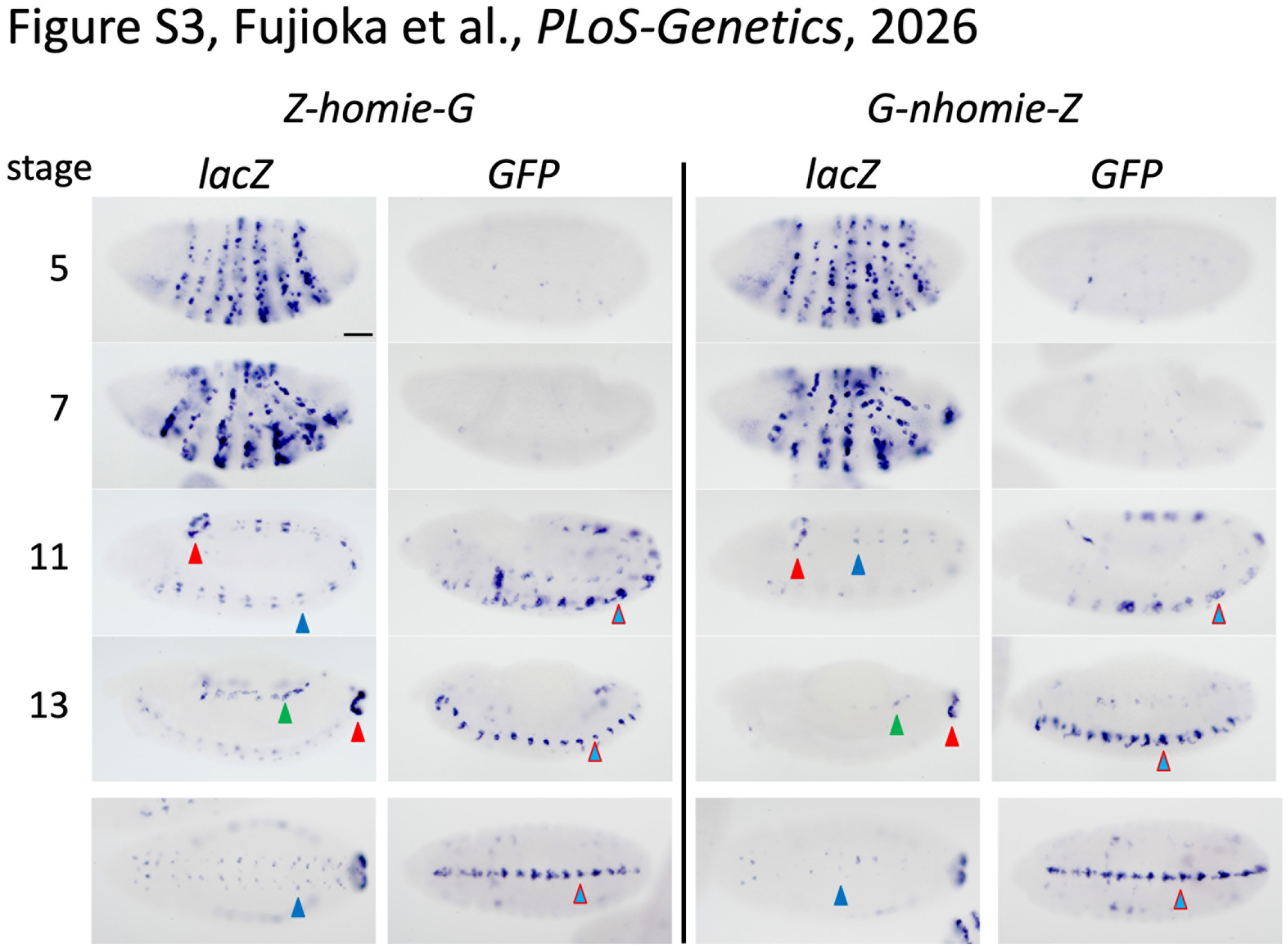

Supplement: S3 Fig — lacZ and gfp expression from Z-homie-G and G-nhomie-Z, labeled as in Fig 2. Consistent with previous studies [17, 26], LR pairing is biased toward one transgenic reporter or the other, depending on the orientation of homie or nhomie in the transgene (see main text). Scale bar: 50μm. (TIF) [file pgen.1011940.s003.tif]

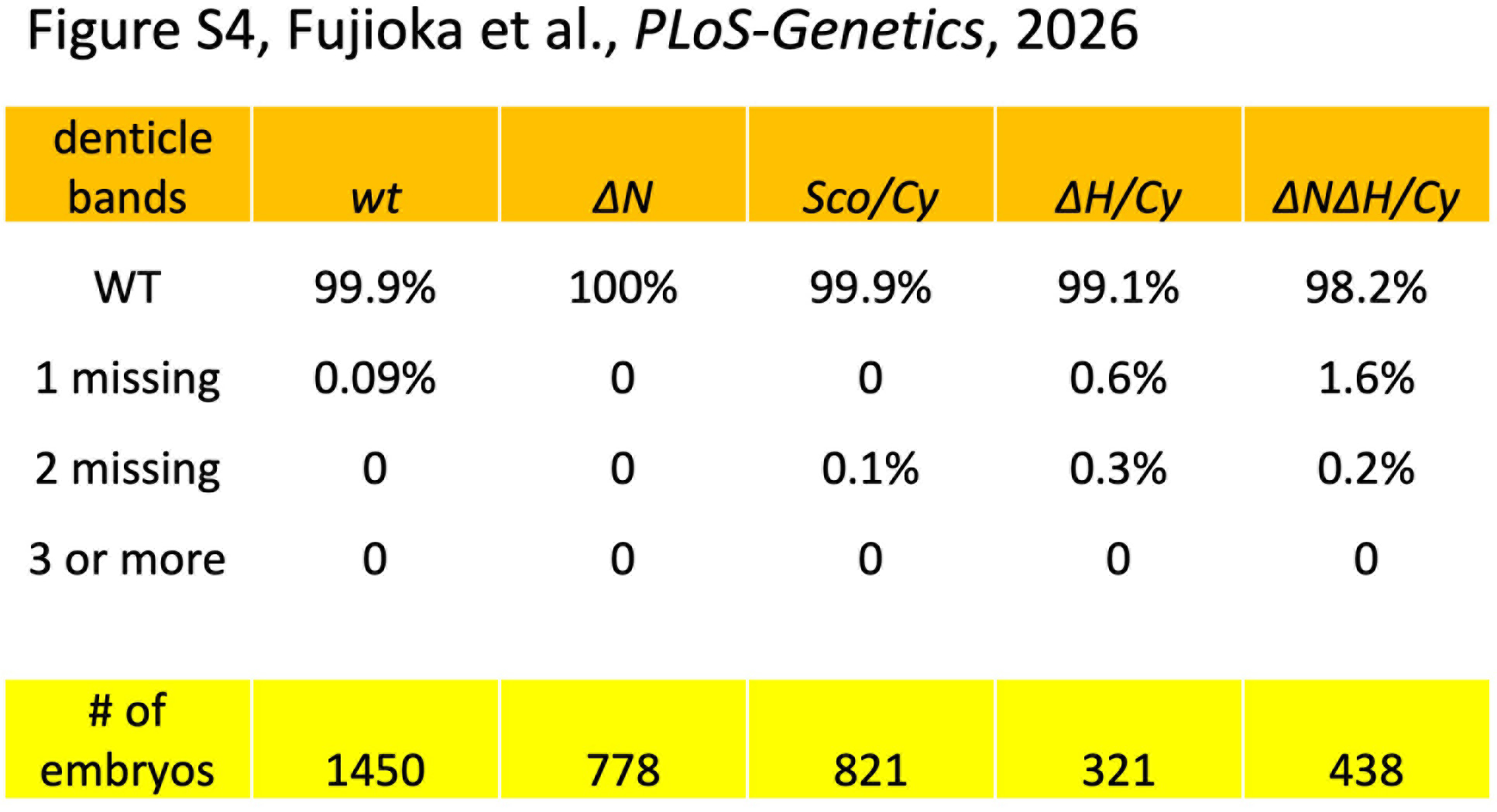

Supplement: S4 Fig — Lines of wt, ∆N, Sco/Cy, ∆H/Cy, and ∆N∆H/Cy were self-crossed, and cuticle defects were counted as either WT (no missing denticle bands), or as having 1, 2, 3, or more missing denticle bands. For ∆H and ∆N∆H, homozygotes are expected to be 25% of the population. Total numbers of counted embryos are shown at the bottom. Number of cuticle preparations included: WT and ∆N: n = 4; Sco/Cy, ∆H/Cy, and ∆N∆H/Cy: n = 3. (TIF) [file pgen.1011940.s004.tif]

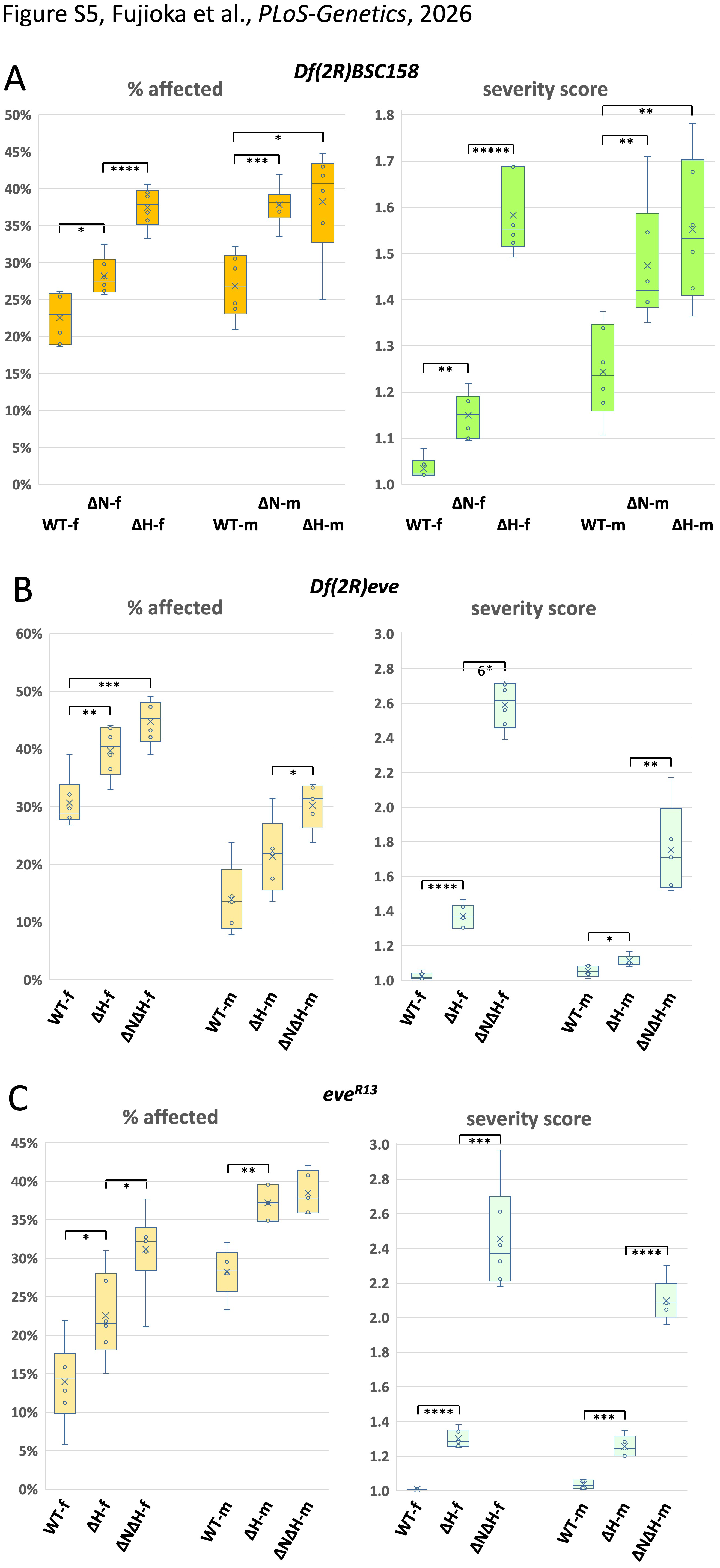

Supplement: S5 Fig — Embryonic cuticle defects were tabulated from the following crosses: A. Sco/Cy (WT), ∆N/Cy (∆N), and ∆H/Cy (∆H) crossed with Df(2R)BSC158/Cy. B. Sco/Cy (WT), ∆H/Cy (∆H), and ∆N∆H/Cy (∆N∆H) crossed with Df(2R)eve/Cy. C. Sco/Cy (WT), ∆H/Cy (∆H), and ∆N∆H/Cy (∆N∆H) crossed with eveR13/Cy. The parental source of the chromosome analyzed is indicated as -f (female) or -m (male) after each genotype. The percentage of embryos showing deleted ventral abdominal denticle bands (left graph, % affected) and the average number of denticle bands deleted per non-wild-type embryo (right graph, severity score) are each shown as a box-and-whiskers plot. The pair-wise significances of differences (p-values) are shown as the number of asterisks. *: p < 0.05, **: p < 0.01, ***: p < 0.001, ****: p < 0.0001, *****: p < 10–5, *6: p < 10–6. The number of cuticle preparations included (n) and the total number of embryos counted were as follows: in A, WT-f (n = 6, 2722 embryos), ∆N-f (n = 6, 1676), ∆H-f (n = 6, 3661), WT-m (n = 6, 1119), ∆N-m (n = 6, 1623), ∆H-m (n = 6, 1000); in B, WT-f (n = 6, 2457), ∆H-f (n = 6, 3773), ∆N∆H-f (n = 6, 2624), WT-m (n = 5, 2123), ∆H-m (n = 5, 2320), ∆N∆H-m (n = 5, 2217); in C, WT-f (n = 6, 1457), ∆H-f (n = 6, 3434), ∆N∆H-f (n = 6, 1500), WT-m (n = 5, 2641), ∆H-m (n = 5, 2939), ∆N∆H-m (n = 5, 3289). (TIF) [file pgen.1011940.s005.tif]

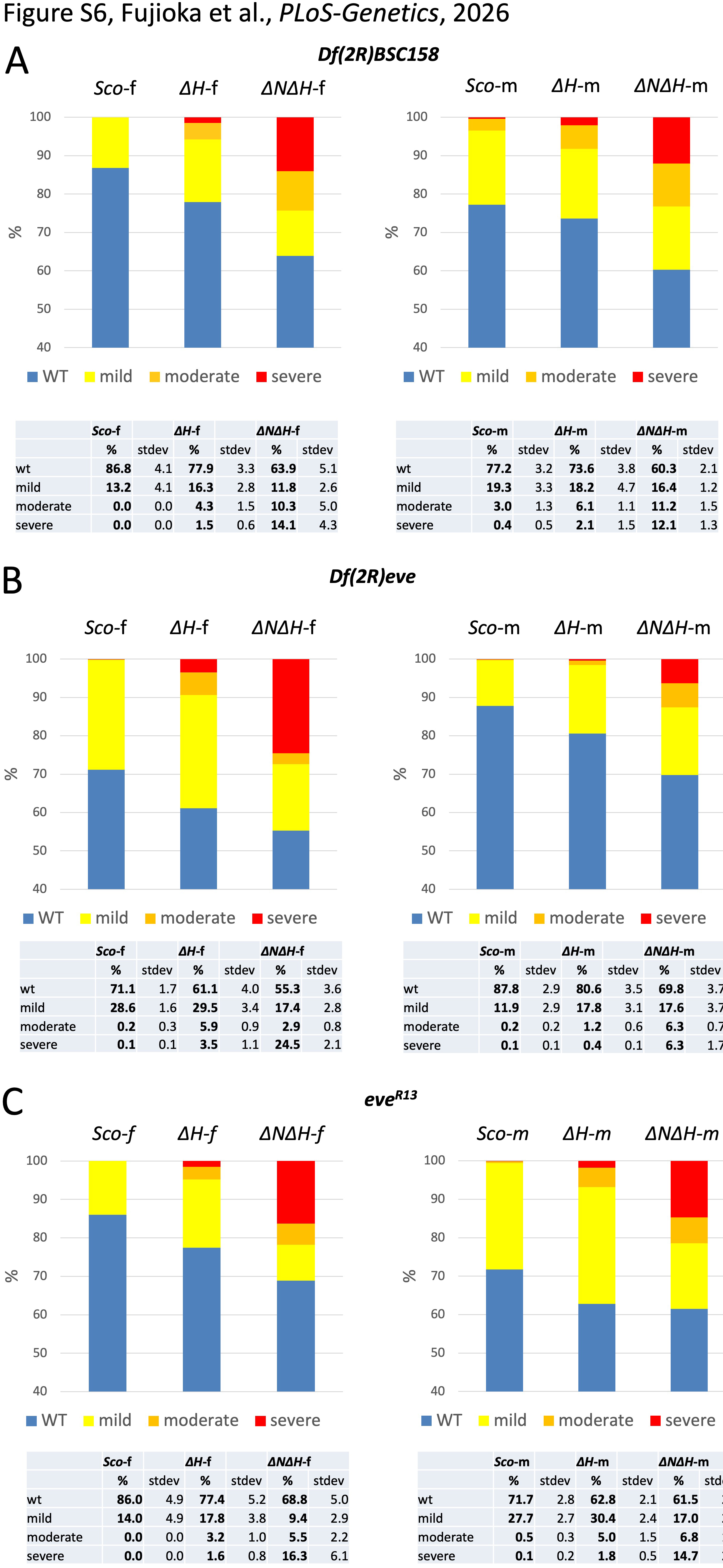

Supplement: S6 Fig — Sco (wild type control from Sco/Cy stock), ∆H/Cy (∆H), and ∆N∆H/Cy (∆N∆H) were crossed with either, in A, Df(2R)BSC158/Cy, or in B, Df(2R)eve/Cy, or in C, eveR13/Cy. The same data used for Figs 5, S5B, and S5C are shown here as stacked graphs. WT: no missing ventral denticle bands; mild: 1 missing; moderate: 2 missing; severe: 3–4 missing. Tables at the bottom show the average percentages of embryos in each deficiency class (%) and their standard deviations (stdev). Scale bar: 50μm. (TIF) [file pgen.1011940.s006.tif]

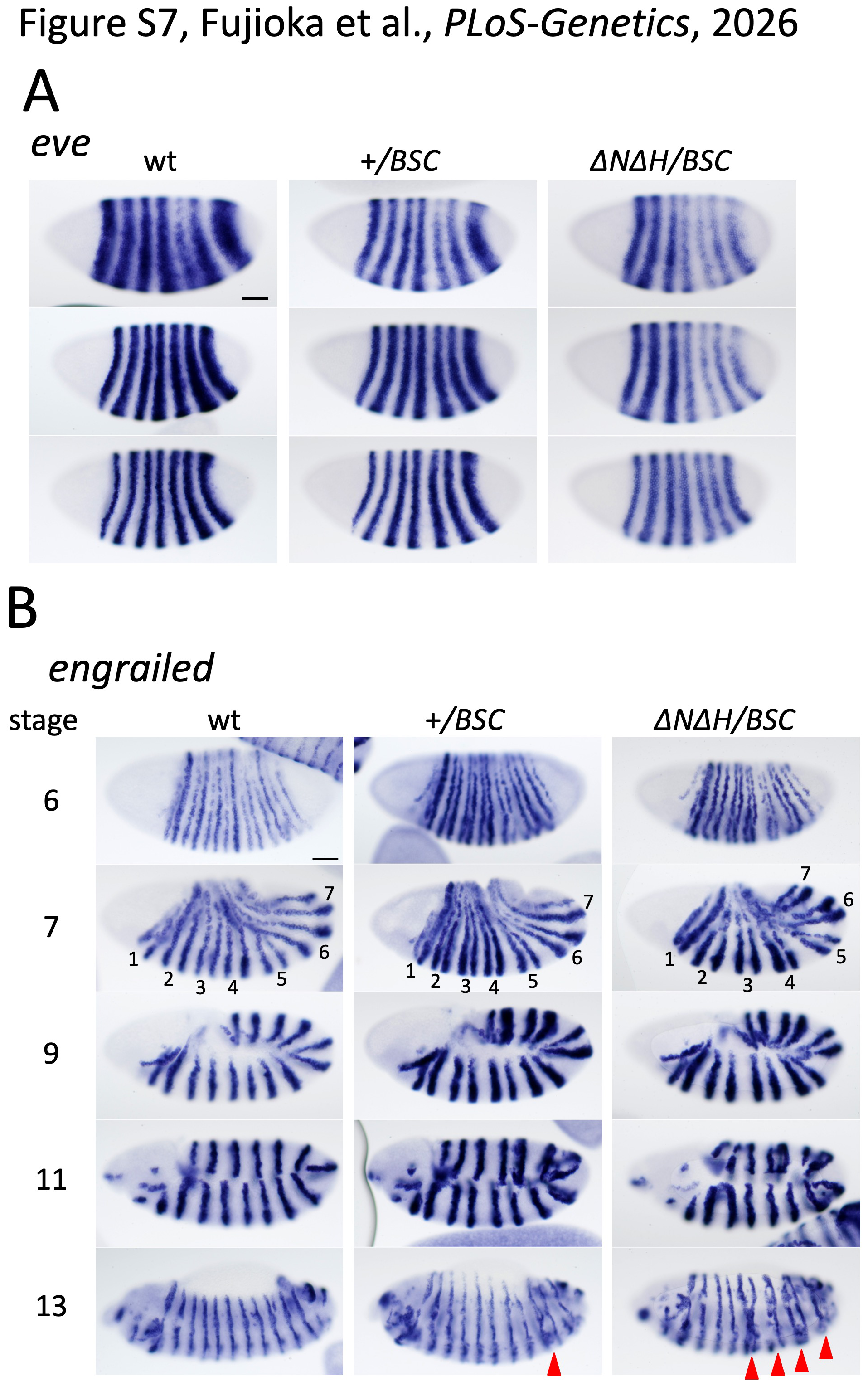

Supplement: S7 Fig — A. eve expression at stage 5 (early to later stage 5 embryos are shown from top to bottom). B. engrailed expression at stages 6, 7, 9, 11, and 13. At stage 7, the positions of early eve stripes are numbered. Fused engrailed stripes that likely prefigure missing denticle bands are marked with red arrowheads at stage 13. wt: homozygous wild-type genotype (both copies of the eve locus are intact), + /BSC: wild type over Df(2R)BSC158 (1 copy of the wild-type eve locus present), ∆N∆H/BSC: ∆N∆H over Df(2R)BSC158 (1 copy of the ∆N∆H eve locus present). Scale bar: 50μm. (TIF) [file pgen.1011940.s007.tif]
